# Supplementary material for: Developing and validating subjective and objective risk-assessment measures for predicting mortality after major surgery: An international prospective cohort study
Source: PLoS Med. 2020 Oct 15;17(10):e1003253. doi: 10.1371/journal.pmed.1003253 (PMC7561094; doi:10.1371/journal.pmed.1003253)
Supplement: S3 Text — (DOCX) [file pmed.1003253.s003.docx]

**S3 Text**

Case Record Form

## Section I: To be completed during surgery

1.1. Hospital number / patient label: ____________________

1.2. Patient surname: ____________________

1.3. Patient first name: ____________________

1.4. DOB (DD/MM/YY): _ _ / _ _ / _ _

1.5. Gender: M ☐ / F ☐

1.6. PostCode: _ _ _ _ _ _ _ _

1.7. NHS/CHI/HSC number: ____________________

1.8. Ethnicity (please select one):

| White  ☐ English / Welsh / Scottish / Northern Irish / British  ☐ Irish  ☐ Gypsy or Irish Traveller  ☐ Any other White background | Mixed / Multiple ethnic groups  ☐ White and Black Caribbean  ☐ White and Black African  ☐ White and Asian  ☐ Any other Mixed / Multiple ethnic background | Asian / Asian British  ☐ Indian  ☐ Pakistani  ☐ Bangladeshi  ☐ Chinese  ☐ Any other Asian background |
| --- | --- | --- |
| Black / African / Caribbean / Black British  ☐ African  ☐ Caribbean  ☐ Any other Black / African / Caribbean background | Other ethnic group  ☐ Arab  ☐ Any other ethnic group, please describe:  ______________________ |  |

2.1. Surgery start (incision) date (DD/MM/YY): _ _ / _ _ / _ _

2.2. Surgery start (incision) time (please select one of the following time periods):

08:00 – 11:59hrs ☐ 12:00 – 15:59hrs ☐ 16:00 – 19:59hrs ☐

20:00 – 23:59hrs ☐ 00:00 – 04:00hrs ☐ 04:00 – 07:59hrs ☐

2.4. Which of these best describes where the patient has come from for this operation?

Home ☐ Inpatient ☐

2.4a. What level of support was the patient receiving on arrival to the operating theatre/anaesthetic room?

Level 0 ☐ Level 1 ☐ Level 2 ☐ Level 3 ☐

2.5. Date of admission to this hospital (DD/MM/YY): _ _ / _ _ / _ _

2.6. Planned operation: (free-text)

2.7. Did the patient have a preoperative assessment before hospital admission?

Y ☐ N ☐ Not applicable (Non-elective admission) ☐

2.8. Operative urgency: Elective ☐ Expedited ☐ Urgent ☐ Immediate ☐

3.1. ASA-PS: I ☐ II ☐ III ☐ IV ☐ V ☐

3.2. Past Medical History (tick all that apply, alternatively select “None of the above”):

Coronary artery disease Y ☐ N ☐

Congestive cardiac failure Y ☐ N ☐

Cancer within last 5 years Y ☐ N ☐

Metastatic cancer (current) Y ☐ N ☐

Stroke / TIA Y ☐ N ☐

Dementia Y ☐ N ☐

COPD Y ☐ N ☐

Pulmonary fibrosis Y ☐ N ☐

Liver Cirrhosis Y ☐ N ☐

End-stage Renal Disease* Y ☐ N ☐

*(eGFR <15 or dialysis-dependent)

Complex polytrauma Y ☐ N ☐

None of the above ☐

3.3. Diabetes:

☐ Not diabetic ☐ Type 1

☐ Type 2 (on insulin) ☐ Type 2 (Diet controlled only)

☐ Type 2 (Non-insulin glucose lowering medication)

3.4. Drug treatments (that the patient would normally be taking)

Diuretic treatment? Y ☐ N ☐ Anti-anginal treatment? Y ☐ N ☐

Digoxin therapy? Y ☐ N ☐ Any anti-hypertensive treatment? Y ☐ N ☐

Warfarin? Y ☐ N ☐ Other treatment-dose anticoagulation? Y ☐ N ☐

Clinical findings

3.5. Body Mass Index: Don’t know ☐ Value if known: _________

3.6. Elevated JVP? Y ☐ N ☐

3.7. Peripheral oedema? Y ☐ N ☐

3.8. Glasgow Coma Scale pre-induction of anaesthesia: _________

3.9. Pre-anaesthetic induction systolic BP: _________

3.10. Pre-anaesthetic induction pulse rate: _________

3.11. Dyspnoea? None ☐ On exertion ☐ limiting activities ☐ At rest ☐

Investigations (within 3 months of surgery)

3.12. Creatinine: Not done ☐ Value if known: _______µmol/L

3.13. Urea: Not done ☐ Value if known: _______mmol/L

3.14. Hb: Not done ☐ Value if known: _______g/L

3.15. Na: Not done ☐ Value if known: _______mmol/L

3.16. K: Not done ☐ Value if known: _______mmol/L

3.17. White cell count: Not done ☐ Value if known: _______x 10^9^cells/L

3.18. HBA1c *: Not done ☐ Value if known: _______mmol/mol

*(IFCC units)

3.19. ECG findings:

☐ Not done ☐ AF 60-90 ☐ Q waves

☐ >4 ectopics ☐ ST or T wave changes ☐ AF >90

☐ Normal ECG ☐ Any other abnormal rhythm

3.20. Radiological findings

No chest X-ray or scan done prior to surgery ☐

Chest X-ray or scan done prior to surgery, and:

Normal appearances seen Y ☐ N ☐

Consolidation seen Y ☐ N ☐

Cardiomegaly seen Y ☐ N ☐

Other abnormality seen Y ☐ N ☐

3.21. Grade of most senior anaesthetist present:

Consultant ☐ Staff & Associate Specialist ☐

ST3-7 trainee or Trust grade equivalent ☐

Core/Foundation year trainee or Trust grade equivalent ☐

3.22. Grade of most senior surgeon present:

Consultant ☐ Staff & Associate Specialist ☐

ST3-7 trainee or Trust grade equivalent ☐

Core/Foundation trainee or Trust grade equivalent ☐

3.23. What is the estimate of the perioperative team of the risk of death within 30days?

<1% ☐ 1-2.5% ☐ 2.6-5% ☐ 5.1-10% ☐ 10.1-50% ☐ >50% ☐

3.24. What has this mortality estimate been based on? (tick all that apply)

Clinical judgment ☐

ASA-PS score ☐

Duke / other Activity status Index ☐

Six-minute walk test or incremental shuttle walk test ☐

Cardiopulmonary exercise testing ☐

Formal frailty assessment (e.g. Edmonton Frail Scale) ☐

Surgical Risk Scale ☐

Surgical Outcome Risk Tool (SORT) ☐

EuroSCORE ☐

POSSUM ☐

P-POSSUM ☐

Surgery specific POSSUM (e.g. Vasc-POSSUM) ☐

Other risk scoring system (please state): __________________________________________

3.25. Has this patient previously had this surgery cancelled/rescheduled?

Y ☐ N ☐ Not known ☐

3.25a. If surgery previously cancelled/rescheduled, what was the reason?

No beds ☐ Clinical reasons ☐ Not known ☐

Other (please describe) ☐: __________________________________________

3.26. Does the perioperative team think that this patient requires critical care after their operation?

Y ☐ N ☐

3.27. Has this patient been referred for postoperative critical care?

Y ☐ N ☐

3.28. For what reason has this patient been referred for postoperative critical care:

Not referred for postoperative critical care ☐

Routine for this type of surgery in this hospital ☐

High risk patient based on preoperative risk stratification ☐

Other: please state

## Section II: To be completed at the end of surgery:

4.1. Surgery end date (DD/MM/YY): _ _ / _ _ / _ _

4.2. Surgery end time (please select one of the following time periods):

08:00 – 11:59hrs ☐ 12:00 – 15:59hrs ☐ 16:00 – 19:59hrs ☐

20:00 – 23:59hrs ☐ 00:00 – 04:00hrs ☐ 04:00 – 07:59hrs ☐

4.3. Anaesthetic technique (select all that apply):

General ☐ Sedation (deep) ☐ Sedation (light) ☐

Epidural ☐ Spinal ☐ Combined spinal/epidural ☐

Regional(Non-neuraxial) ☐ Local infiltration ☐

4.4. Have there been any critical / unexpected events perioperatively?

Y ☐ N ☐ If yes – please describe (free-text)

­­­­­­­

4.5. In the past 30 days, how many procedures have been performed (including this one)?

1 ☐ 2 ☐ >2 ☐

4.6. Estimated total blood loss: 0-100ml ☐ 101-500ml ☐ 501-999ml ☐ ≥1000ml ☐

4.7. Was there peritoneal contamination?

Not applicable ☐ No soiling ☐ Minor soiling ☐

Local pus ☐ Free bowel content, pus or blood ☐

4.8. Was the procedure for Malignancy?

Not malignant ☐ Primary Malignancy only ☐

Malignancy + nodal metastases ☐ Malignancy + distal metastases ☐

4.9. Actual operation: (free-text)

4.10. Immediate postoperative destination:

Recovery ☐ ICU/HDU ☐ PACU/OIR ☐

4.11. If critical care admission planned but patient going to recovery, please state reasons why:

N/A (patient not planned for critical care admission) ☐

No bed currently available: planned ICU/HDU/PACU/OIR admission later today ☐

No bed available – will be going to normal ward after recovery ☐

PACU/OIR/ICU/HDU care no longer clinically necessary ☐

The routine pathway in this hospital is theatre 🡪 recovery 🡪 Critical Care ☐

Other: please state:

## Section III: Day 7 review

5.1. Is the patient still alive and in hospital on postoperative Day 7? Y ☐ N ☐

5.2. If No –

What was the date of hospital discharge (DD/MM/YY)? _ _ / _ _ / _ _

5.3. If discharged, what was their status at discharge?

Alive ☐ Dead ☐ Not known ☐

5.4. If Alive –

Has the patient returned to their preoperative level of mobility?

Y ☐ N ☐ Not known ☐

5.5. Is there a non-clinical reason for remaining in hospital? (e.g. awaiting social services, residential placement etc.) Y ☐ N ☐

**Thank you. If the patient remains in hospital, please complete section IV. If they have been discharged from hospital or died before day 7 please put a line through section 4.**

## Section IV: Day 7 Post-Operative Morbidity Survey

**Please tick all that apply. If discharged from hospital before D7, please draw a line through this page.**

6.1. Is there a new requirement for:

- O2 therapy? Y ☐ N ☐
- Ventilatory support? Y ☐ N ☐

6.2a. Has the patient developed a temperature of >38 in the past 24h? Y ☐ N ☐

6.2b. Is the patient currently on antibiotics? Y ☐ N ☐

6.3a. Has the patient passed <500ml urine in the past 24h? Y ☐ N ☐

6.3b. Does the patient have a raised serum creatinine (>30% from pre-operative level)?

Y ☐ N ☐

6.3c. Is a urinary catheter in situ for non-surgical/anatomical reasons? Y ☐ N ☐

6.4. Has the patient had diagnostic tests and /or treatment for any of the following in the past 24 hours:

- New myocardial infarction or ischaemia Y ☐ N ☐
- Hypotension (requiring IV fluid >200ml/h or drug therapy) Y ☐ N ☐
- Atrial or ventricular arrhythmias Y ☐ N ☐
- Cardiogenic pulmonary oedema Y ☐ N ☐
- Thrombotic event requiring anticoagulation Y ☐ N ☐

6.5a. Is the patient unable to tolerate enteral diet (either food or tube feeding) for any non-surgical reason including nausea, vomiting and abdominal distension? Y ☐ N ☐

6.5b. Has there been administration of an anti-emetic in the past 24h? Y ☐ N ☐

6.6. Is there a new:

- focal neurological deficit Y ☐ N ☐
- confusion Y ☐ N ☐
- delirium Y ☐ N ☐
- coma (associated with administration of sedation) Y ☐ N ☐
- coma (not sedation related) Y ☐ N ☐

6.7. Has there been a requirement for any of the following within the past 24 hours

- Packed erythrocytes Y ☐ N ☐
- Fresh frozen plasma, platelets or cryoprecipitate Y ☐ N ☐

6.8. Has there been:

- a wound dehiscence requiring surgical exploration Y ☐ N ☐
- drainage of pus from the operation wound with/without isolation of organisms

Y ☐ N ☐

6.9. Does the patient have post-operative pain significant enough to require:

- parenteral opioids Y ☐ N ☐
- regional analgesia Y ☐ N ☐

## Section V: To be completed 60 days postoperatively

7.1. Did the patient have a **planned** ICU/HDU/PACU/OIR admission **on** the day of surgery?

Y ☐ N ☐

7.2. Did the patient have an **unplanned** ICU/HDU/PACU/OIR admission **on** the day of surgery?

Y ☐ N ☐

7.3. Did the patient have an **unplanned** postoperative ICU/HDU admission **after** day of surgery?

Y ☐ N ☐

7.4. Is the patient still in hospital? (Primary admission after surgery)

Y ☐ N ☐

7.5. If not, what was the date of hospital discharge (DD/MM/YY)? _ _ / _ _ / _ _

7.6. If discharged, what was their status at discharge?

Alive ☐ Dead ☐ N/A: Remains in-patient at 60d post-op ☐

7.7. Number of days spent in critical care after surgery: _ _ _

**Thank you for completing this form.**

**We are grateful for your support for the SNAP-2: EPICCS study**

**The online study data entry system can be accessed here:**

<https://snap2.snapresearch.org.uk/>

**If you would like updates on the study, please refer to the study website:**

<http://www.niaa-hsrc.org.uk/SNAP-2>
