# Supplementary material for: Developing and validating subjective and objective risk-assessment measures for predicting mortality after major surgery: An international prospective cohort study
Source: PLoS Med. 2020 Oct 15;17(10):e1003253. doi: 10.1371/journal.pmed.1003253 (PMC7561094; doi:10.1371/journal.pmed.1003253)
Supplement: S4 Text — (DOCX) [file pmed.1003253.s004.docx]

**S4 Text: Continuous NRI analysis and reclassification tables**

In continuous NRI analysis, two model predictions are compared. The net proportion of events (deaths) reclassified correctly plus the net proportion of non-events (survivors) reclassified correctly is calculated.

A correct reclassification is a concordant prediction to observed reality, and can either be: 1) an increase in predicted risk (“Up”) in a patient who goes on to die; or 2) a decrease in predicted risk (“Down”) in a patient who goes on to survive· The converse is true for an incorrect reclassification, i.e. a discordant pair of prediction to reality: 1) an increased in predicted risk in a patient who goes on to survive; or 2) a decrease in predicted risk in a patient who goes on to die.

Expressed mathematically:

$\mathrm{NRI}_{\mathrm{continuous}}=P\left( \mathrm{Up} | \mathrm{Event} \right)-P\left( \mathrm{Down} | \mathrm{Event} \right)+P\left( \mathrm{Down} | Non-event \right)-P\left( \mathrm{Up} | Non-event \right)$

We therefore computed the risk predictions according to subjective clinical assessment, SORT and the logistic regression model with predictor variables combining information from both. Supplementary Table 1 shows the net reclassification improvement calculation for SORT vs· subjective clinical assessment, and Supplementary Table 2 shows the net reclassification improvement calculation for the combined model vs· subjective clinical assessment.

*Continuous net reclassification table for SORT vs. clinical assessments*

|  | Down | Up |
| --- | --- | --- |
| Survived | 12480 | 5177 |
| Died | 126 | 62 |
|  |  |  |

$\mathrm{NRI}_{\mathrm{continuous}}=P\left( \mathrm{Up} | \mathrm{Event} \right)-P\left( \mathrm{Down} | \mathrm{Event} \right)+P\left( \mathrm{Down} | Non-event \right)-P\left( \mathrm{Up} | Non-event \right)$

Therefore,

$$\mathrm{NRI}_{\mathrm{continuous}}=\frac{62}{126+62}-\frac{126}{126+62}+\frac{12480}{12480+5177}-\frac{5177}{12480+5177}=0.073$$

*Continuous net reclassification table for Combined Model (SORT + clinical assessments) vs. clinical assessments alone*

|  | Down | Up |
| --- | --- | --- |
| Survived | 17579 | 78 |
| Died | 175 | 13 |

$\mathrm{NRI}_{\mathrm{continuous}}=P(Up|Event)-P(Down|Event)+P(Down|Non-event)-P(Up|Non-event)$

Therefore,

$$\mathrm{NRI}_{\mathrm{continuous}}=\frac{13}{175+13}-\frac{175}{175+13}+\frac{17579}{17579+78}-\frac{78}{17579+78}=0.1295$$
