# Supplementary material for: Developing and validating subjective and objective risk-assessment measures for predicting mortality after major surgery: An international prospective cohort study
Source: PLoS Med. 2020 Oct 15;17(10):e1003253. doi: 10.1371/journal.pmed.1003253 (PMC7561094; doi:10.1371/journal.pmed.1003253)
Supplement: S5 Text — (DOCX) [file pmed.1003253.s005.docx]

**S3 Text**

**Patient characteristics of sub-groups used in sensitivity analyses**

We performed a number of sensitivity analyses, each with a different cohorts and sub-cohorts of patients drawn from the original study (n = 26,216) dataset with complete data variables:

- The first consisted of the full sample of patients with complete data, including those undergoing obstetric surgery who were excluded from the main study.
- The second consisted of patients with a higher risk profile. The inclusion criteria for this sub-group was consistent with those used in the METS study led by Wijeysundera *et al* investigating the performance of subjective clinical assessment compared to other objective measures in predicting functional capacity before major surgery. For this group, we included patients who had one or more of the following: intermediate-to-high risk surgery (intra-peritoneal, intra-thoracic, or major vascular surgery); history of coronary artery disease; history of heart failure; history of previous cerebrovascular disease; history of diabetes mellitus require drug therapy; preoperative renal insufficiency; history of peripheral arterial disease; history of hypertension; age ≥70 years
- The third consisted of patients who received subjective clinical assessments of risk that were informed by other information sources in addition to clinical judgement alone
- The fourth used the main patient dataset, but stratified by country (Australia/New Zealand vs. UK)
- The fifth used the main patient dataset but excluded those with any missing P-POSSUM variables.
- The sixth assessed the performance of the combined model (subjective clinical assessment + SORT objective risk prediction) in 7 specialty sub-cohorts from the overall dataset.

S1 Table shows the patient characteristics for the sensitivity analysis groupings, alongside the patient characteristics of the overall patient dataset.
