# Supplementary material for: Developing and validating subjective and objective risk-assessment measures for predicting mortality after major surgery: An international prospective cohort study
Source: PLoS Med. 2020 Oct 15;17(10):e1003253. doi: 10.1371/journal.pmed.1003253 (PMC7561094; doi:10.1371/journal.pmed.1003253)
Supplement: S6 Text — (DOCX) [file pmed.1003253.s006.docx]

**S6 Text**

**Sensitivity Analysis Number 1**

In the first sensitivity analysis, after repeating the main study analyses using the full cohort of patients available from SNAP-2: EPICCS, including those undergoing obstetric procedures, there were minimal differences seen to our main study findings. The SORT was again the best-calibrated of the pre-existing models in this larger cohort, and all objective risk tools again over-predicted risk (S1 Figure A-C; Hosmer-Lemeshow p-values all <0.0001 for the SORT, P-POSSUM, and SRS). The estimates for AUROC were minimally affected (Figure 2D; AUROC SORT=0.91 (95% confidence interval (CI): 0.90–0.93); P-POSSUM=0.90, (95% CI: 0.88–0.92); SRS=0.85 (95% CI: 0.83–0.88). The AUROC for the SORT was still significantly better than SRS (p <0.0001), but not P-POSSUM (p = 0.121). Subjective assessment in this first sensitivity analysis demonstrated similar over-prediction of risk (S1 Fig, Hosmer-Lemeshow test p <0.001) but similar discrimination (S1 Fig AUROC = 0.89, 95% CI: 0.87–0.92) to the main study analysis. Differences in discrimination between subjective assessment and SORT were again not significantly different (95% CI for difference in AUROC: -0.02 to -0.01, p = 0.216). Continuous NRI analysis again did not show improvement in classification when using the SORT compared to subjective assessment.
