# Supplementary material for: Developing and validating subjective and objective risk-assessment measures for predicting mortality after major surgery: An international prospective cohort study
Source: PLoS Med. 2020 Oct 15;17(10):e1003253. doi: 10.1371/journal.pmed.1003253 (PMC7561094; doi:10.1371/journal.pmed.1003253)
Supplement: S7 Text — (DOCX) [file pmed.1003253.s007.docx]

**S7 Text: Sensitivity Analysis 2**

For the second sensitivity analysis in the higher-risk patient sub-group, the performance of P-POSSUM, SRS and SORT are similar to their performance in the original patient dataset. The calibration plots of the risk tools (Supplementary Figure S3A-C) appear similar to the calibration plots obtained in the main study analysis, and again tended to over-predict risk. The Hosmer-Lemeshow chi-squared goodness-of-fit test showed a significant deviation from the line of unity for all three models (p-value <0·001 all three models). All risk prediction models exhibited poorer discrimination than in the main study analysis (S3 Fig Plot D), however the rank order of their discrimination performance was unchanged· SORT again performed the best in this sub-group with an AUROC of 0·88 (95% confidence interval: 0·86–0·90), followed by P-POSSUM (AUROC = 0·86, 95% CI: 0·84–0·89) and SRS (AUROC = 0·81, 95% CI: 0·78–0·84).

Subjective clinical assessment in this sub-group again demonstrated a tendency to over-predict risk on calibration plot analysis (S3 Fig plot E, Hosmer-Lemeshow test p <0·001). The clinician mortality predictions also demonstrated good discrimination (S3 Fig Plot F, AUROC = 0·85, 95% CI: 0·82–0·89) in this sub-group. The subjective assessment discrimination in this sub-group was not significantly different to the main study analysis cohort (p = 0·155).

The findings of this sensitivity analysis suggest that performance of subjective clinical assessment and all the objective risk models is poorer in a higher risk cohort. However, the relative performance of the models in comparison to each other, and in comparison with subjective clinical assessment, was consistent with our main study findings.
