# Supplementary material for: Developing and validating subjective and objective risk-assessment measures for predicting mortality after major surgery: An international prospective cohort study
Source: PLoS Med. 2020 Oct 15;17(10):e1003253. doi: 10.1371/journal.pmed.1003253 (PMC7561094; doi:10.1371/journal.pmed.1003253)
Supplement: S8 Text — (DOCX) [file pmed.1003253.s008.docx]

**S8 Text: Sensitivity Analysis 3**

For this third sensitivity analysis, the performance of subjective clinical assessment was assessed in the sub-group of patients who received subjective risk estimates informed by other sources in addition to clinical judgement. The calibration of these assessments appeared similar to that in the main study analysis, and subjective clinical assessment again appeared to over-predict risk (Supplementary Figure S4). Subjective clinical assessment discrimination performance in this sub-group was equivalent to the performance obtained in main study findings (AUROC = 0·88, 95% CI: 0·85–0·91, p = 0·551).
