# Supplementary material for: Developing and validating subjective and objective risk-assessment measures for predicting mortality after major surgery: An international prospective cohort study
Source: PLoS Med. 2020 Oct 15;17(10):e1003253. doi: 10.1371/journal.pmed.1003253 (PMC7561094; doi:10.1371/journal.pmed.1003253)
Supplement: S9 Text — (DOCX) [file pmed.1003253.s009.docx]

**S9 Text: Sensitivity Analysis 4**

For the fourth sensitivity analysis, we separately repeated the external validation of the objective risk tools in the UK and then the Australian/New Zealand subsets of patients (Supplementary Figure S5). P-POSSUM, SRS and SORT all over-predicted risk in both subsets of data and their calibration curves appeared similar to those obtained in the main analysis· While the absolute number of events (deaths within 30 days) in the Australian/New Zealand cohort was small (n = 34) and the proportion of deaths (1·09%) was lower than the UK (Supplementary Table S3), this difference in proportions was not statistically significant (p = 0·245). There were however wide confidence intervals seen for the proportion of deaths within each bin of the calibration curves for Australia/New Zealand. The calibration for SORT in Australian/New Zealand cases was visibly worse than the calibration in the UK cohort. AUROCs for the objective risk tools in the Australia/New Zealand subset was not significantly different to the AUROCs in the UK subset (Supplementary Table S4).

Similarly, we repeated the evaluation of subjective clinical assessments in these two patient subsets (Supplementary Figure S6), and the calibration of subjective assessments was comparable in both. AUROCs for subjective assessments in both subsets likewise did not show significant differences (Supplementary Table S4).
