# Supplementary material for: Developing and validating subjective and objective risk-assessment measures for predicting mortality after major surgery: An international prospective cohort study
Source: PLoS Med. 2020 Oct 15;17(10):e1003253. doi: 10.1371/journal.pmed.1003253 (PMC7561094; doi:10.1371/journal.pmed.1003253)
Supplement: S10 Text — (DOCX) [file pmed.1003253.s010.docx]

**S10 Text: Sensitivity Analysis 5**

For the fifth sensitivity analysis, we compared results for patients who did and did not have complete P-POSSUM data, to evaluate whether our approach of single value imputation for missing P-POSSUM data was appropriate. Using the subgroup of patients who had complete P-POSSUM variables (n = 18,362, see Supplementary Table S3 for patient characteristics), patients with complete P-POSSUM variables appeared to be older, have higher ASA-PS grades, and underwent higher severity surgery in comparison with those with missing P-POSSUM variables, The AUROC for clinical assessments in the subgroup with full P-POSSUM variables was 0.896, which was not significantly different to the AUROC obtained for clinical assessments in the main study analysis (p = 0.587), and the predictions were similarly calibrated to clinical assessments in the main study analysis, again with a tendency to over-predict risk (Supplementary Figure S7 for calibration plots). When comparing the performance of P-POSSUM (AUROC = 0.893), SRS (AUROC = 0.838), and SORT (AUROC = 0.899) in this subgroup, the performance was again similar to that of the main study cohort (p >0.05 for all comparisons).
