# Supplementary material for: Developing and validating subjective and objective risk-assessment measures for predicting mortality after major surgery: An international prospective cohort study
Source: PLoS Med. 2020 Oct 15;17(10):e1003253. doi: 10.1371/journal.pmed.1003253 (PMC7561094; doi:10.1371/journal.pmed.1003253)
Supplement: S11 Text — (DOCX) [file pmed.1003253.s011.docx]

**S11 Text: Sensitivity Analysis 6**

*A sixth sensitivity analysis was performed by testing the performance of the combined model incorporating subjective clinical assessment with SORT-predicted risk in different specialty subgroups. Performance of the combined model was good in all subgroups. AUROC ranged from 0.865 in cardiothoracic surgery to 0.946 in gynaecology/urology surgery patients. Calibration was acceptable in all subgroups except for vascular surgery patients.*
