# Supplementary material for: Developing and validating subjective and objective risk-assessment measures for predicting mortality after major surgery: An international prospective cohort study
Source: PLoS Med. 2020 Oct 15;17(10):e1003253. doi: 10.1371/journal.pmed.1003253 (PMC7561094; doi:10.1371/journal.pmed.1003253)
Supplement: S1 Table — ASA-PS, American Society of Anesthesiologists Physical Status; COPD, Chronic Obstructive Pulmonary Disease; IQR, interquartile range; P-POSSUM, Portsmouth-Physiology and Operative Severity Score for the enUmeration of Mortality; SORT, Surgical Outcome Risk Tool; SRS, Surgical Risk Scale. (DOCX) [file pmed.1003253.s013.docx]

**S1 Table**

*Sensitivity analyses. Characteristics of the patient subgroups used in all sensitivity analyses. The restricted cohort used in the first sensitivity analysis was older, had higher ASA-PS grades, higher proportions of Xmajor or Complex surgery, higher incidences of comorbid disease and a higher mortality rate compared to the whole cohort of patients used in the main study analyses. The subgroup with clinical assessments made in conjunction with other tools was similar in characteristics to the whole cohort of patients used in the main analyses. The Australian and New Zealand cohort had a lower proportion of obstetric surgery (and consequently a higher proportion of males) and a higher proportion of minor/intermediate surgery, but a comparable percentage mortality.*

|  | 1: Overall cohort | 2: Restricted subgroup of higher risk patients | 3: Subgroup of patients with clinical assessments made in conjunction with other tools | 4: UK cohort | 4: Aus/NZ cohort | 5: Full P-POSSUM variables | 5: Missing P-POSSUM variables |
| --- | --- | --- | --- | --- | --- | --- | --- |
| N | 26216 | 12985 | 4751 | 19503 | 3128 | 18362 | 8140 |
| Sex = M (%) | 10676 (40.7) | 6429 (49.5) | 2407 (50.7) | 9035 (46.3) | 1636 (52.3) | 8258 (45.0) | 2555 (31.4) |
| Age (median [IQR]) | 57 [37, 72] | 71 [61, 79] | 68 [55, 77] | 62 [46, 74] | 59 [42, 71] | 63 [46, 74] | 39 [30, 62] |
| Operative Urgency (%) |  |  |  |  |  |  |  |
| Elective | 13469 (51.4) | 7108 (54.7) | 2367 (49.8) | 10681 (54.8) | 1380 (44.1) | 9269 (50.5) | 4295 (52.8) |
| Expedited | 3596 (13.7) | 1899 (14.6) | 722 (15.2) | 2603 (13.3) | 708 (22.6) | 2740 (14.9) | 905 (11.1) |
| Urgent | 7802 (29.8) | 3625 (27.9) | 1468 (30.9) | 5678 (29.1) | 939 (30.0) | 5650 (30.8) | 2262 (27.8) |
| Immediate | 1349 ( 5.1) | 353 ( 2.7) | 194 ( 4.1) | 541 ( 2.8) | 101 ( 3.2) | 697 ( 3.8) | 669 ( 8.2) |
| ASA-PS Class (%) |  |  |  |  |  |  |  |
| I | 6439 (24.6) | 656 ( 5.1) | 504 (10.6) | 3944 (20.2) | 518 (16.6) | 3161 (17.2) | 3322 (40.9) |
| II | 11672 (44.5) | 6020 (46.4) | 1729 (36.4) | 8964 (46.0) | 1228 (39.3) | 8278 (45.2) | 3469 (42.7) |
| III | 6696 (25.5) | 5312 (40.9) | 1935 (40.7) | 5559 (28.5) | 1015 (32.4) | 5653 (30.8) | 1121 (13.8) |
| IV | 1339 ( 5.1) | 955 ( 7.4) | 560 (11.8) | 983 ( 5.0) | 354 (11.3) | 1181 ( 6.4) | 189 ( 2.3) |
| V | 70 (0.3) | 42 ( 0.3) | 23 ( 0.5) | 53 ( 0.3) | 13 ( 0.4) | 58 ( 0.3) | 15 ( 0.2) |
| Procedure Severity (%) |  |  |  |  |  |  |  |
| Minor | 2120 (8.1) | 879 ( 6.8) | 244 ( 5.1) | 1588 ( 8.1) | 363 (11.6) | 1410 ( 7.7) | 730 ( 9.0) |
| Intermediate | 4897 (18.7) | 2393 (18.4) | 637 (13.4) | 3720 (19.1) | 803 (25.7) | 3249 (17.7) | 1689 (20.8) |
| Major | 10507 (40.1) | 4042 (31.1) | 1436 (30.2) | 6498 (33.3) | 980 (31.3) | 6475 (35.3) | 4127 (50.8) |
| Xmajor | 5287 (20.2) | 3703 (28.5) | 1186 (25.0) | 4790 (24.6) | 491 (15.7) | 4326 (23.6) | 1001 (12.3) |
| Complex | 3405 (13.0) | 1968 (15.2) | 1248 (26.3) | 2907 (14.9) | 491 (15.7) | 2870 (15.7) | 576 ( 7.1) |
| Surgical specialty (%) |  |  |  |  |  |  |  |
| Gastrointestinal surgery | 4472 (17.1) | 3399 (26.2) | 1325 (27.9) | 3931 (20.2) | 541 (17.3) | 3742 (20.4) | 769 ( 9.5) |
| Gynaecology/Urology | 4309 (16.4) | 2039 (15.7) | 629 (13.2) | 3886 (19.9) | 423 (13.5) | 3187 (17.4) | 1166 (14.4) |
| Neuro/Spinal surgery | 1208 (4.6) | 538 ( 4.1) | 180 ( 3.8) | 1002 ( 5.1) | 206 ( 6.6) | 1010 ( 5.5) | 211 ( 2.6) |
| Obstetrics | 3578 (13.7) | 0 ( 0.0) | 0 ( 0.0) | 0 ( 0.0) | 0 ( 0.0) | 898 ( 4.9) | 2706 (33.3) |
| Orthopaedics | 6772 (25.8) | 4262 (32.8) | 1231 (25.9) | 6053 (31.0) | 719 (23.0) | 5153 (28.1) | 1673 (20.6) |
| Thoracic/Cardiac surgery | 1033 (3.9) | 386 ( 3.0) | 609 (12.8) | 839 ( 4.3) | 194 ( 6.2) | 895 ( 4.9) | 152 ( 1.9) |
| Vascular | 674 (2.6) | 625 ( 4.8) | 224 ( 4.7) | 540 ( 2.8) | 134 ( 4.3) | 572 ( 3.1) | 108 ( 1.3) |
| Other | 4163 (15.9) | 1736 (13.4) | 553 (11.6) | 3252 (16.7) | 911 (29.1) | 2868 (15.7) | 1336 (16.5) |
| Past Medical History: Coronary Artery Disease (%) | 3032 (11.6) | 2623 (20.2) | 1069 (22.5) | 2522 (12.9) | 507 (16.2) | 2586 (14.1) | 489 ( 6.0) |
| Past Medical History: Congestive Cardiac Failure (%) | 897 (3.4) | 751 ( 5.8) | 346 ( 7.3) | 686 ( 3.5) | 207 ( 6.6) | 772 ( 4.2) | 140 ( 1.7) |
| Past Medical History: Metastatic Cancer (active) (%) | 825 (3.1) | 622 ( 4.8) | 256 ( 5.4) | 682 ( 3.5) | 143 ( 4.6) | 705 ( 3.8) | 130 ( 1.6) |
| Past Medical History: Dementia (%) | 676 (2.6) | 658 ( 5.1) | 207 ( 4.4) | 597 ( 3.1) | 79 ( 2.5) | 596 ( 3.2) | 96 ( 1.2) |
| Past Medical History: COPD (%) | 1957 (7.5) | 1653 (12.7) | 582 (12.3) | 1659 ( 8.5) | 296 ( 9.5) | 1673 ( 9.1) | 318 ( 3.9) |
| Past Medical History: Pulmonary Fibrosis (%) | 180 (0.7) | 2623 (20.2) | 1069 (22.5) | 2522 (12.9) | 507 (16.2) | 2586 (14.1) | 489 ( 6.0) |
| Past Medical History: Diabetes (%) |  |  |  |  |  |  |  |
| Type 1 | 301 (1.1) | 207 ( 1.6) | 46 ( 1.0) | 244 ( 1.3) | 30 ( 1.0) | 237 ( 1.3) | 69 ( 0.8) |
| Type 2 (Dietary-controlled) | 734 (2.8) | 491 ( 3.8) | 186 ( 3.9) | 536 ( 2.7) | 78 ( 2.5) | 555 ( 3.0) | 189 ( 2.3) |
| Type 2 (Insulin-controlled) | 850 (3.2) | 691 ( 5.3) | 202 ( 4.3) | 566 ( 2.9) | 195 ( 6.2) | 688 ( 3.8) | 171 ( 2.1) |
| Type 2 (Oral hypoglycaemic medication) | 1647 (6.3) | 1468 (11.3) | 404 ( 8.5) | 1346 ( 6.9) | 224 ( 7.2) | 1356 ( 7.4) | 316 ( 3.9) |
| No diabetes | 22670 (86.5) | 10124 (78.0) | 3910 (82.4) | 16800 (86.2) | 2599 (83.1) | 15505 (84.5) | 7374 (90.8) |
| Past Medical History: Liver Cirrhosis (%) | 225 (0.9) | 148 ( 1.1) | 62 ( 1.3) | 183 ( 0.9) | 41 ( 1.3) | 199 ( 1.1) | 32 ( 0.4) |
| Past Medical History: Renal Disease (%) | 381 (1.5) | 324 ( 2.5) | 77 ( 1.6) | 284 ( 1.5) | 97 ( 3.1) | 339 ( 1.8) | 44 ( 0.5) |
| Postoperative Length of Stay (median [IQR]) | 2 [1, 5] | 3 [1, 8] | 5 [2, 9] | 3 [1, 5] | 3 [1, 6] | 3 [1, 7] | 2 [1, 3] |
| SORT-calculated mortality risk (median [IQR]) | 0·00 [0.00, 0.01] | 0.01 [0.00, 0.03] | 0.01 [0.00, 0.04] | 0.00 [0.00, 0.01] | 0.01 [0.00, 0.02] | 0.01 [0.00, 0.02] | 0.00 [0.00, 0.01] |
| P-POSSUM-calculated mortality risk (median [IQR]) | 0.01 [0.01, 0.03] | 0.02 [0.01, 0.05] | 0.02 [0.01, 0.07] | 0.01 [0.01, 0.03] | 0.01 [0.00, 0.03] | 0.01 [0.01, 0.03] | 0.01 [0.00, 0.01] |
| SRS-calculated mortality risk (median [IQR]) | 0.02 [0.01, 0.04] | 0.02 [0.01, 0.10] | 0.04 [0.02, 0.10] | 0.02 [0.01, 0.04] | 0.02 [0.01, 0.04] | 0.02 [0.01, 0.04] | 0.01 [0.00, 0.04] |
| 30-day mortality (%) | 317 ( 1.2) | 261 ( 2.0) | 128 ( 2.7) | 283 ( 1.5) | 34 ( 1.1) | 281 ( 1.5) | 41 ( 0.5) |

*ASA-PS = American Society of Anesthesiology Physical Status; COPD = Chronic Obstructive Pulmonary Disease; SORT = Surgical Outcome Risk Tool; IQR = Interquartile Range; P-POSSUM = Portsmouth-Physiology and Operative Severity Score for the enUmeration of Mortality; SRS = Surgical Risk Scale.*
