# Supplementary material for: Developing and validating subjective and objective risk-assessment measures for predicting mortality after major surgery: An international prospective cohort study
Source: PLoS Med. 2020 Oct 15;17(10):e1003253. doi: 10.1371/journal.pmed.1003253 (PMC7561094; doi:10.1371/journal.pmed.1003253)
Supplement: S2 Table — (%) represents row percentage. (DOCX) [file pmed.1003253.s014.docx]

**S2 Table**

*Confusion matrix of patients 30-day mortality outcomes vs. clinician predictions. (%) represent row percentages.*

|  |  | **30-day mortality**  **N (%)** | |  |
| --- | --- | --- | --- | --- |
|  | **Total** | **Alive** | **Dead** | **p** |
|  | 17845 | 17657 | 188 |  |
| **Subjective clinician assessment of risk (%)** |  |  |  | <0.001 |
| <1% | 12948 | 12926 (99.8) | 22 (0.2) |  |
| 1-2.5% | 2997 | 2973 (99.2) | 24 (0.8) |  |
| 2.6-5% | 890 | 866 (97.9) | 19 (2.1) |  |
| 5.1-10% | 571 | 544 (95.3) | 27 (4.7) |  |
| 10.1-50% | 312 | 264 (84.6) | 48 (15.4) |  |
| >50% | 132 | 84 (63.6) | 48 (36.4) |  |
