# Supplementary material for: Developing and validating subjective and objective risk-assessment measures for predicting mortality after major surgery: An international prospective cohort study
Source: PLoS Med. 2020 Oct 15;17(10):e1003253. doi: 10.1371/journal.pmed.1003253 (PMC7561094; doi:10.1371/journal.pmed.1003253)
Supplement: S3 Table — We found no significant difference in discrimination using any of the risk prediction tools or using subjective assessment when comparing their performance in the UK and Australian/New Zealand data sets. AUROC, Area Under Receiver Operating Characteristic curve; P-POSSUM, Portsmouth-Physiology and Operative Severity Score for the enUmeration of Mortality; SORT, Surgical Outcome Risk Tool; SRS, Surgical Risk Scale. (DOCX) [file pmed.1003253.s015.docx]

**S3 Table**

*AUROCs of the objective risk tools and subjective assessment, compared between the UK and Australian/New Zealand data subsets. We found no significant difference in discrimination using any of the risk prediction tools or using subjective assessment when comparing their performance in the UK and Australian/New Zealand datasets. P-POSSUM = Portsmouth-Physiology and Operative Severity Score for the enUmeration of Mortality; SRS = Surgical Risk Scale; SORT = Surgical Outcome Risk Tool.*

| **Model** | **Australia/New Zealand** | **UK** | **p-value** |
| --- | --- | --- | --- |
| P-POSSUM | 0·8959 | 0·8923 | 0·8932 |
| SRS | 0·8081 | 0·8509 | 0·1584 |
| SORT | 0·8719 | 0·9042 | 0·2110 |
| Clinical | 0·8796 | 0·8873 | 0·8602 |
