# Supplementary material for: Developing and validating subjective and objective risk-assessment measures for predicting mortality after major surgery: An international prospective cohort study
Source: PLoS Med. 2020 Oct 15;17(10):e1003253. doi: 10.1371/journal.pmed.1003253 (PMC7561094; doi:10.1371/journal.pmed.1003253)
Supplement: S4 Table — (DOCX) [file pmed.1003253.s016.docx]

**S4 Table**

*Discrimination and calibration performance of the new combined prediction model in different specialty subgroups.*

| Specialty subgroup | n | AUROC | 95% CI for AUROC | Hosmer-Lemeshow test statistic | Hosmer-Lemeshow p-value |
| --- | --- | --- | --- | --- | --- |
| Gastrointestinal surgery | 4472 | 0.916 | 0.884 to 0.948 | 8.59 | 0.283 |
| Gynaecology/Urology | 4309 | 0.946 | 0.901 to 0.990 | 7.86 | 0.249 |
| Orthopaedic | 6772 | 0.899 | 0.869 to 0.930 | 6.80 | 0.450 |
| Neuro/Spinal surgery | 1208 | 0.922 | 0.873 to 0.971 | 10.29 | 0.173 |
| Thoracic/Cardiac surgery | 1033 | 0.865 | 0.749 to 0.981 | 9.30 | 0.318 |
| Vascular | 674 | 0.882 | 0.824 to 0.940 | 20.26 | 0.009 |
| Other | 4163 | 0.928 | 0.886 to 0.970 | 8.13 | 0.322 |
