# Supplementary figures and images for: Developing and validating subjective and objective risk-assessment measures for predicting mortality after major surgery: An international prospective cohort study
Source: PLoS Med. 2020 Oct 15;17(10):e1003253. doi: 10.1371/journal.pmed.1003253 (PMC7561094; doi:10.1371/journal.pmed.1003253)

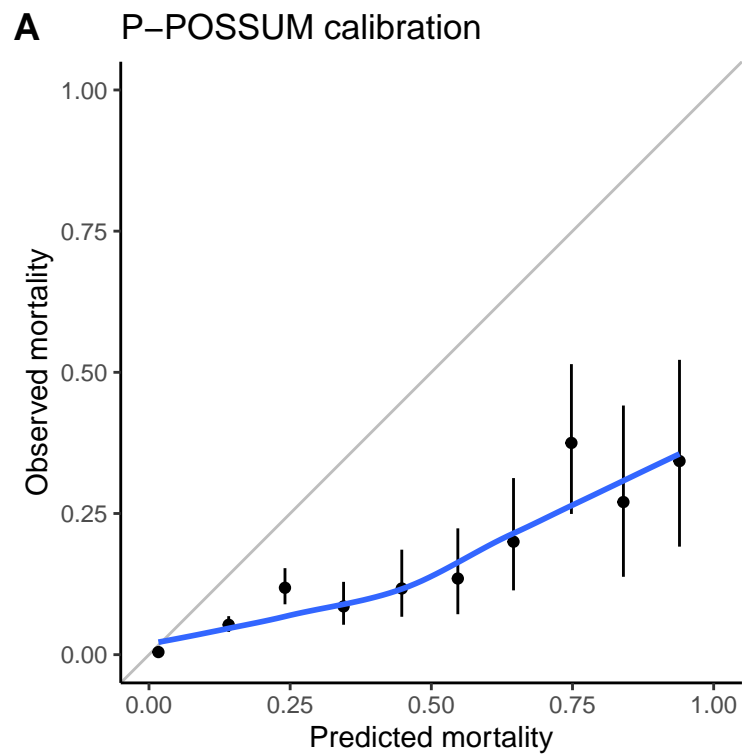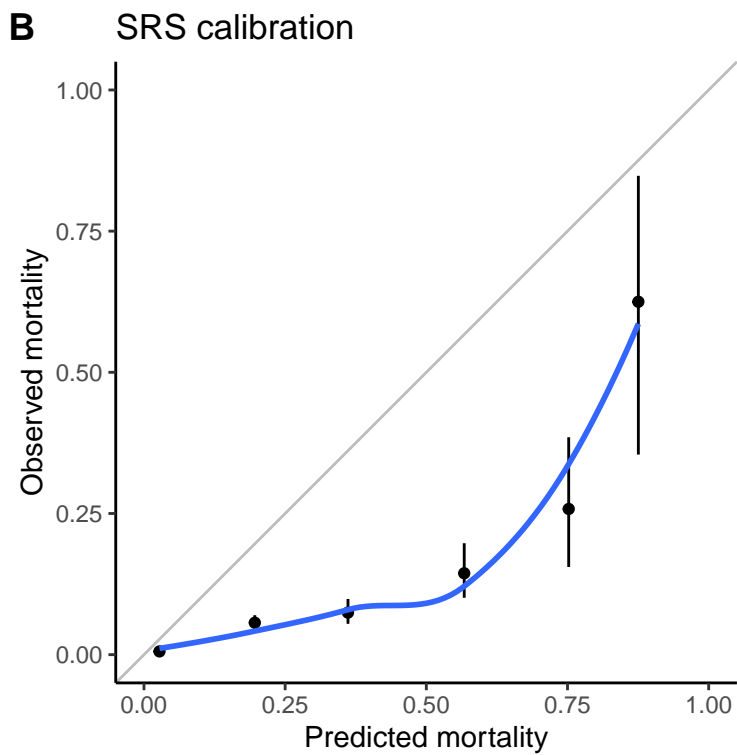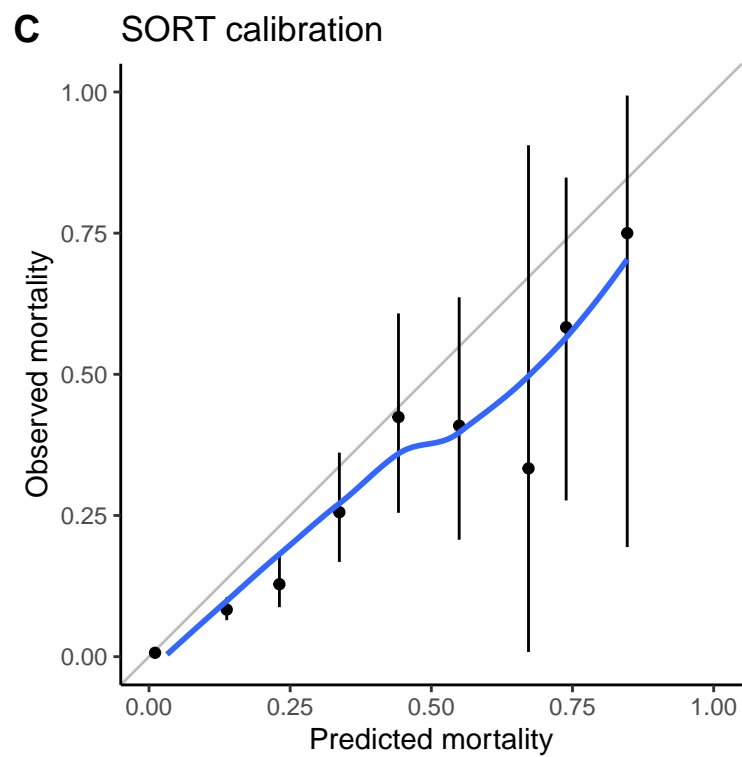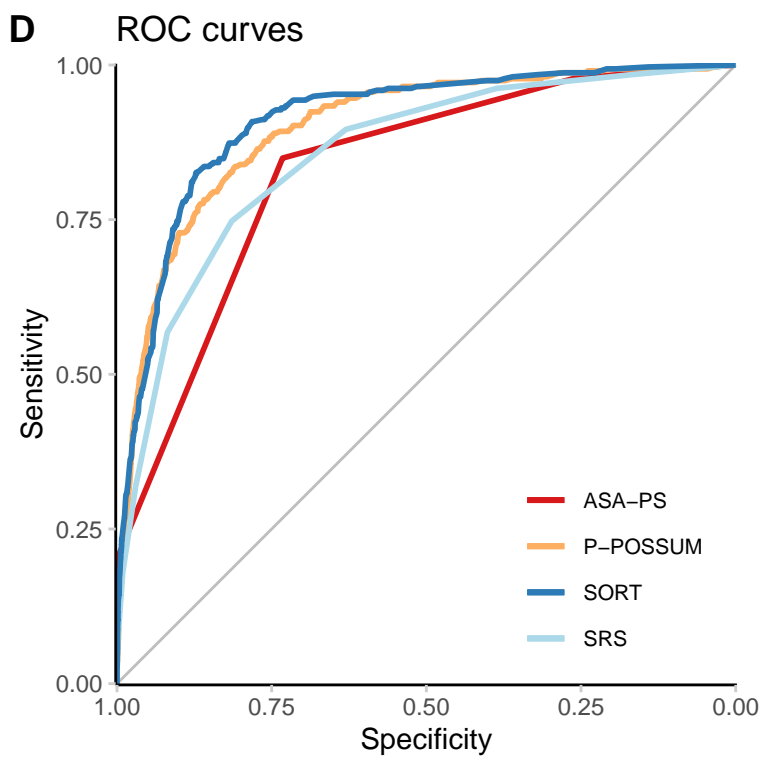

Supplement: S1 Fig — In the calibration plots (A–C), nonparametric smoothed best-fit curves (blue) are shown along with the point estimates for predicted versus observed mortality (black dots) and their 95% CIs (black lines) within each decile of predicted mortality. External validation of all 3 models were performed on the entire SNAP-2: EPICCS patient data set (n = 25,854). CI, confidence interval; P-POSSUM, Portsmouth-Physiology and Operative Severity Score for the enUmeration of Mortality; ROC, Receiver Operating Characteristic; SNAP-2: EPICCS, Second Sprint National Anaesthesia Project: EPIdemiology of Critical Care provision after Surgery; SORT, Surgical Outcome Risk Tool; SRS, Surgical Risk Scale. (PDF) [file pmed.1003253.s017.pdf]

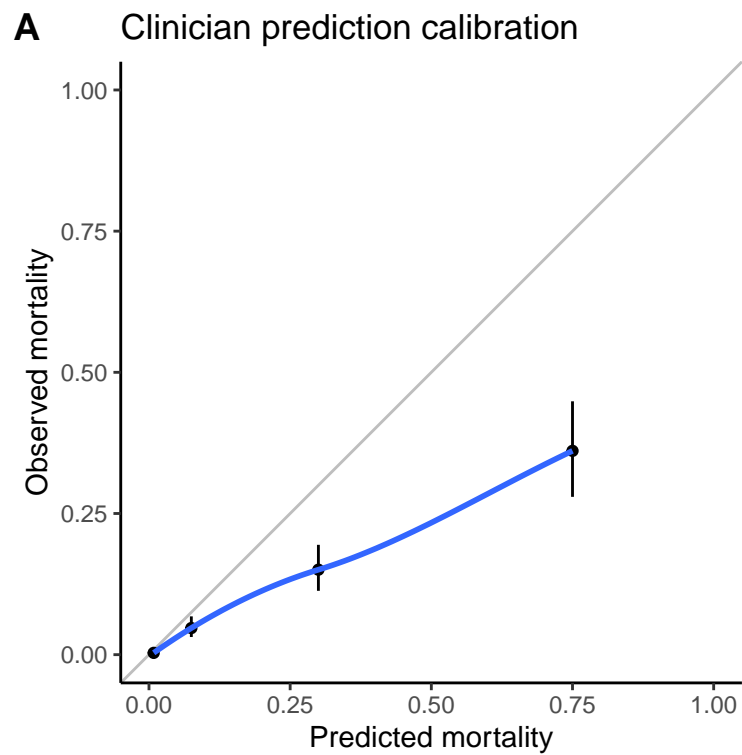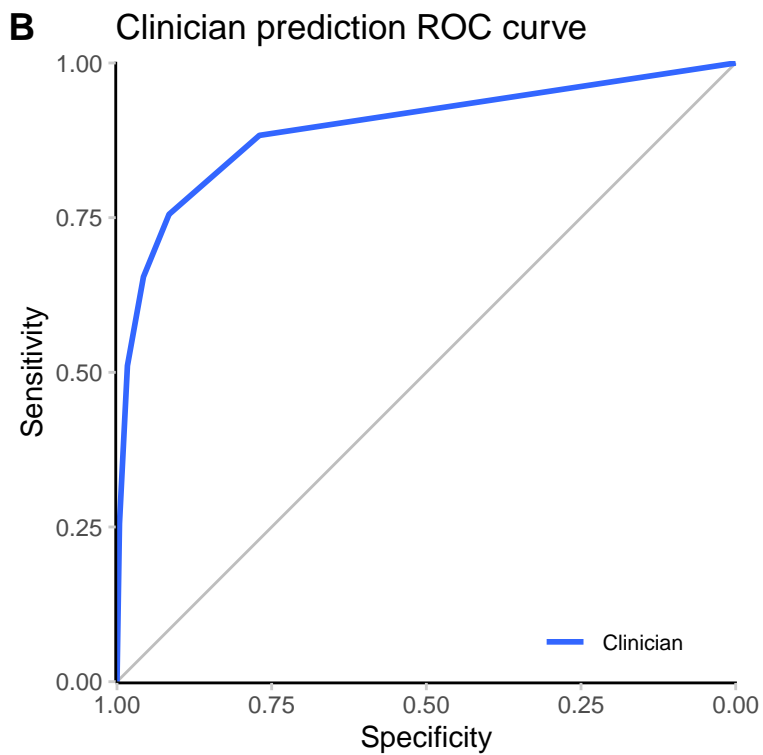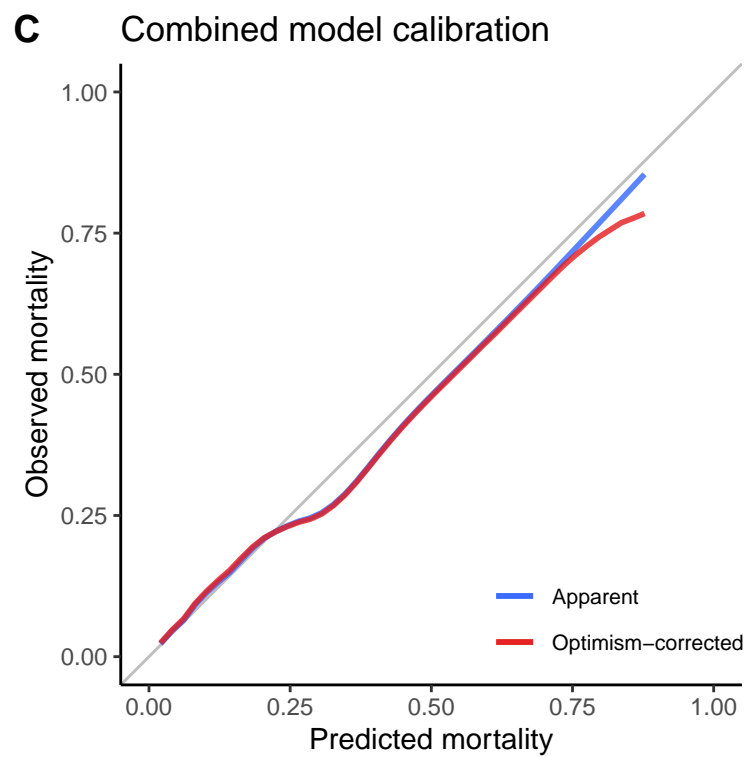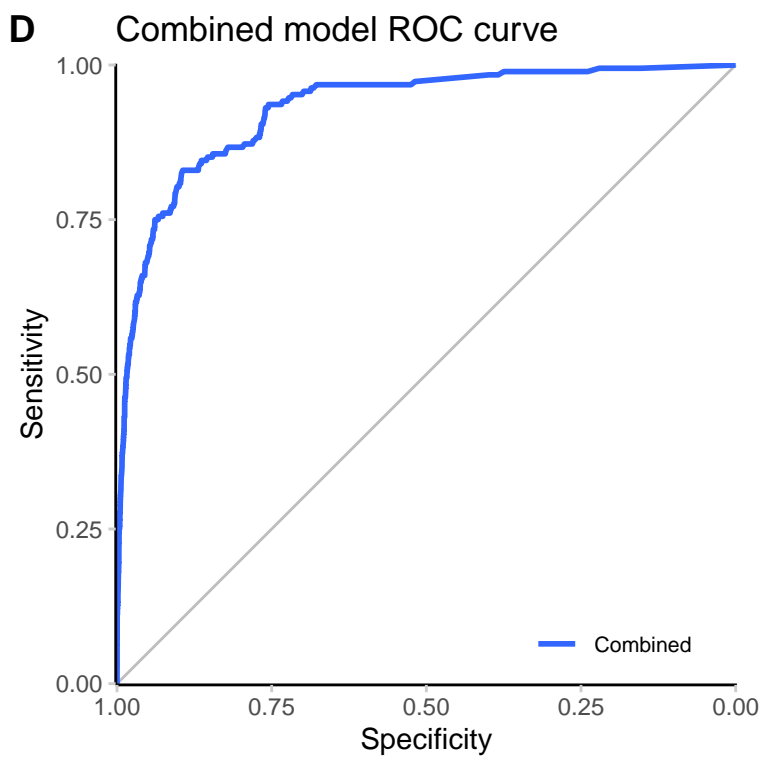

Supplement: S2 Fig — For (A), a nonparametric smoothed best-fit curve (blue) is shown along with the point estimates for predicted versus observed mortality (black dots) and their 95% CIs (black lines) within each range of clinician predicted mortality. For (C), the apparent (blue) and optimism-corrected (red) nonparametric smoothed calibration curves are shown, the latter was generated from 1,000 bootstrapped resamples of the data set. CI, confidence interval; ROC, Receiver Operating Characteristic; SNAP-2: EPICCS, Second Sprint National Anaesthesia Project: EPIdemiology of Critical Care provision after Surgery; SORT, Surgical Outcome Risk Tool. (PDF) [file pmed.1003253.s018.pdf]

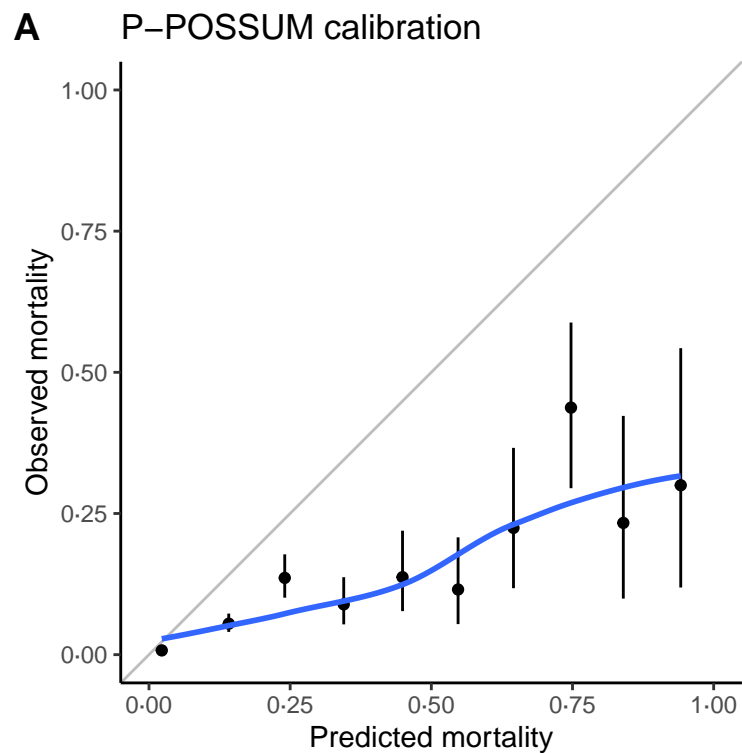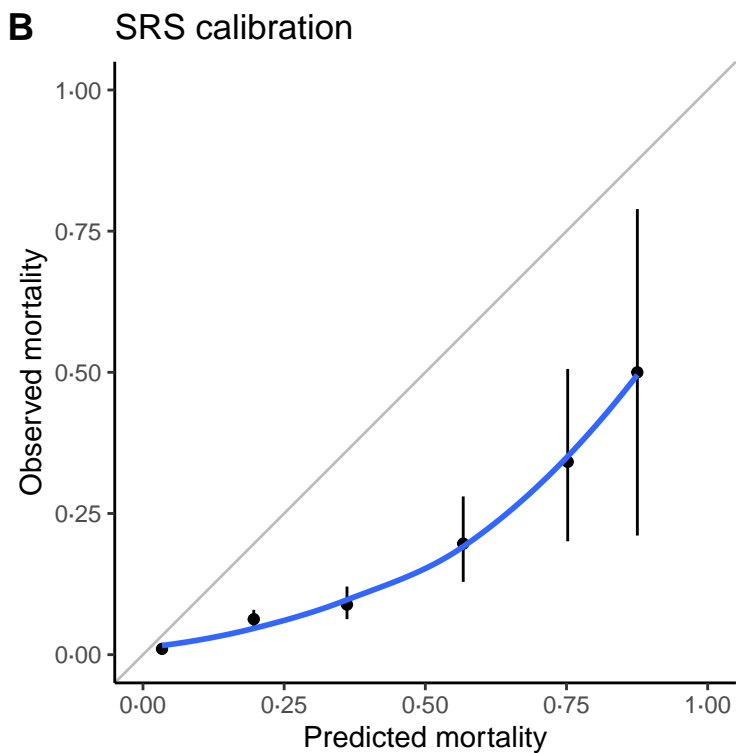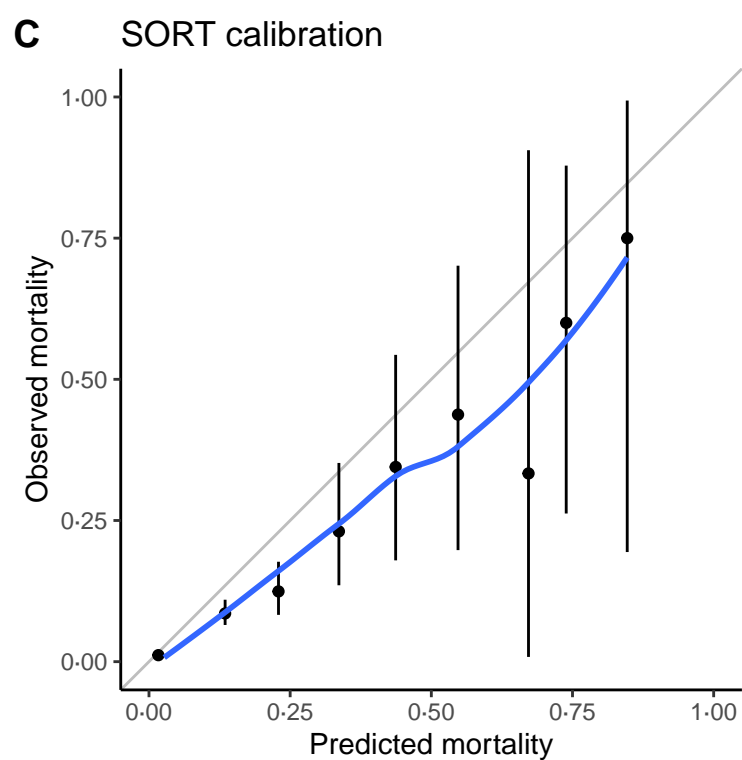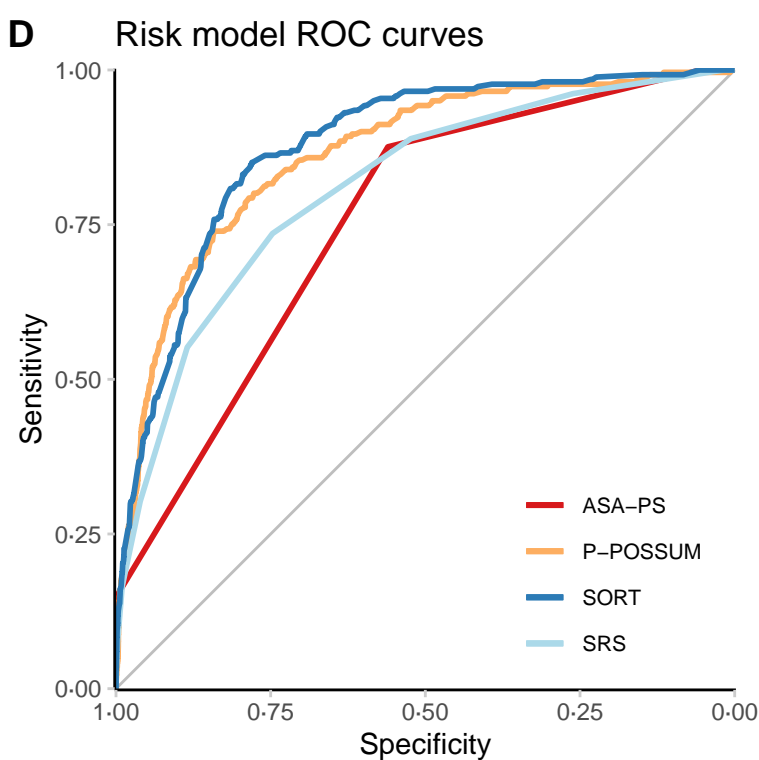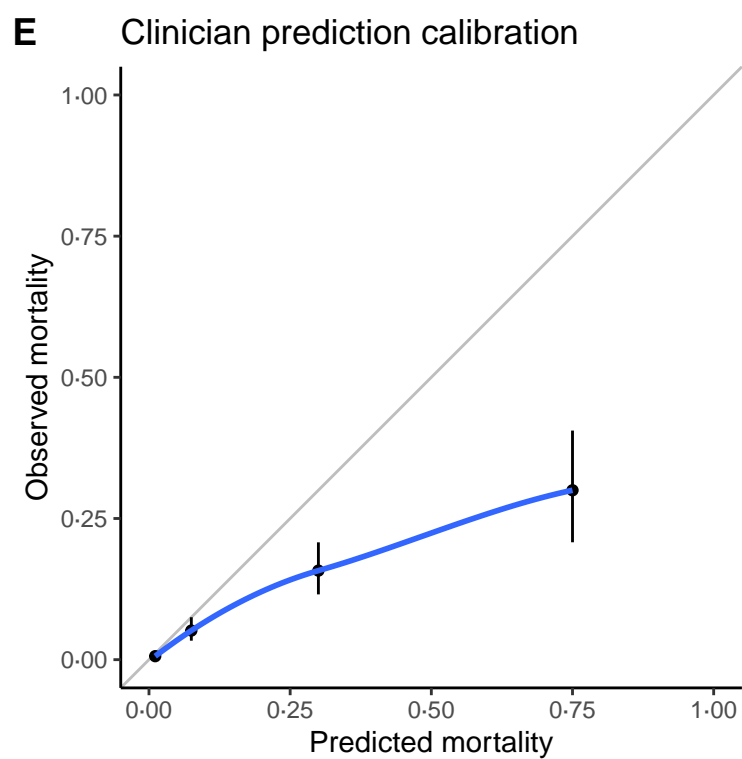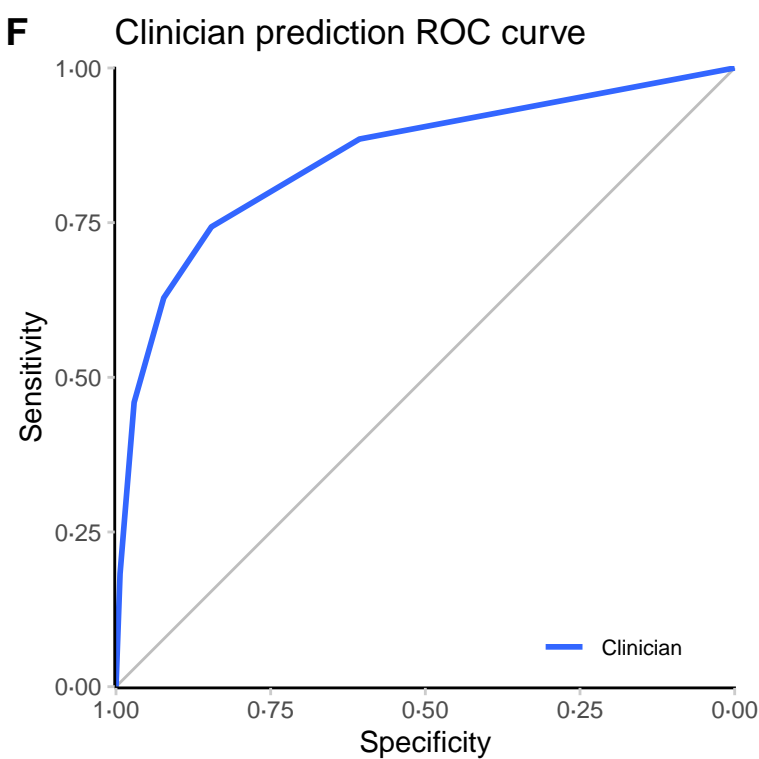

Supplement: S3 Fig — The AUROCs for P-POSSUM, SRS, SORT, and clinical assessments were 0.863, 0.810, 0.875, and 0.853 in this subgroup, respectively. AUROC, Area Under Receiver Operating Characteristic curve; P-POSSUM, Portsmouth-Physiology and Operative Severity Score for the enUmeration of Mortality; SORT, Surgical Outcome Risk Tool; SRS, Surgical Risk Scale. (PDF) [file pmed.1003253.s019.pdf]

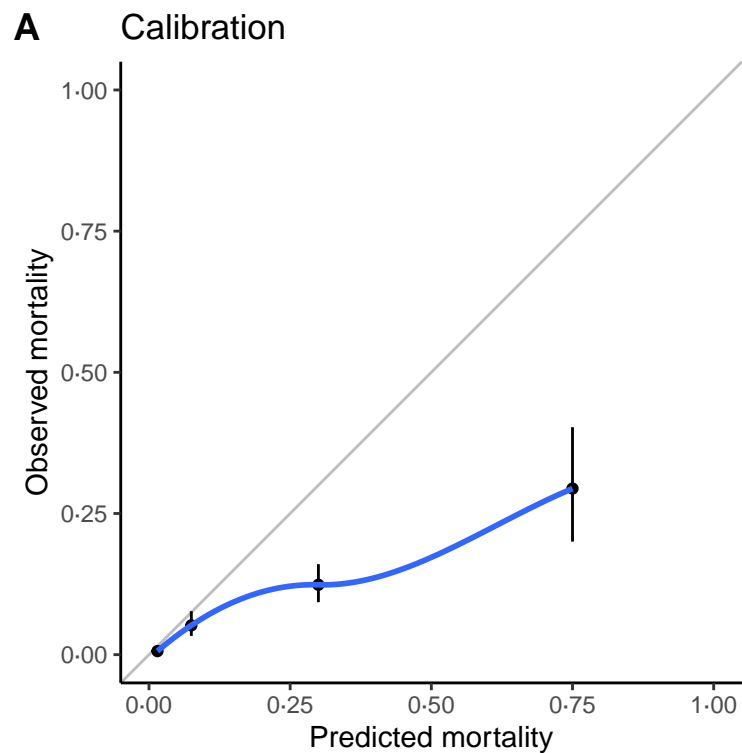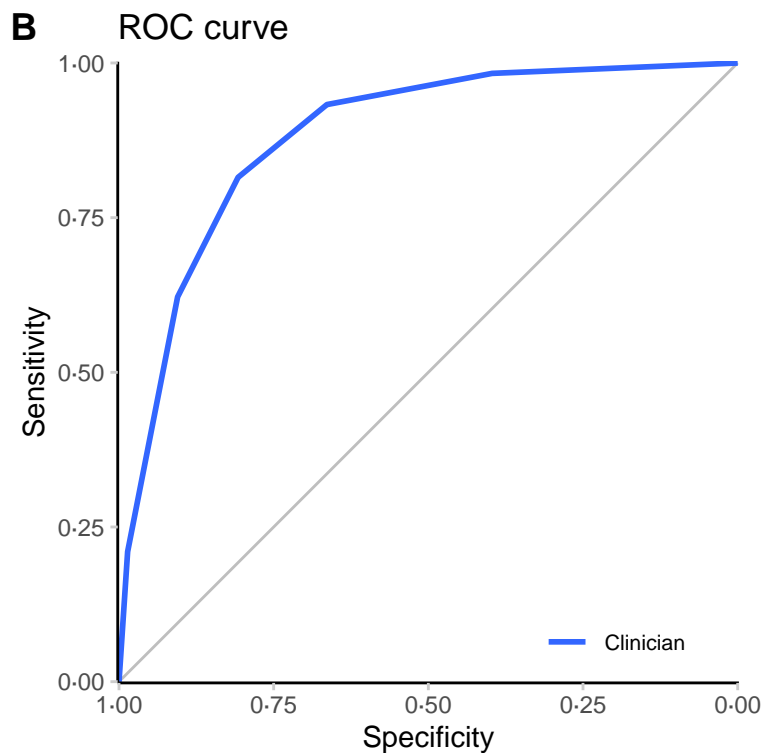

Supplement: S4 Fig — The AUROC for clinical assessments was 0.880 in this subgroup. AUROC, Area Under Receiver Operating Characteristic curve. (PDF) [file pmed.1003253.s020.pdf]

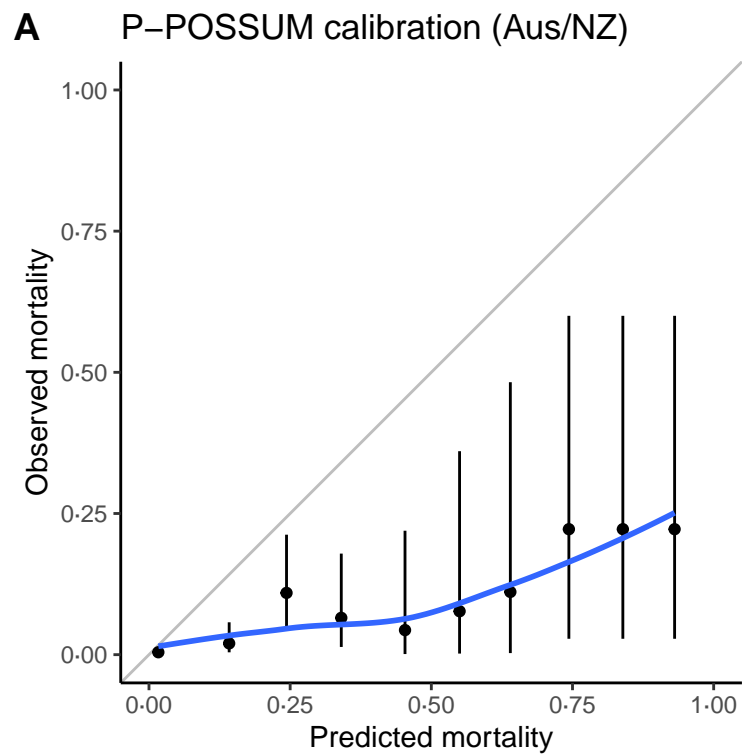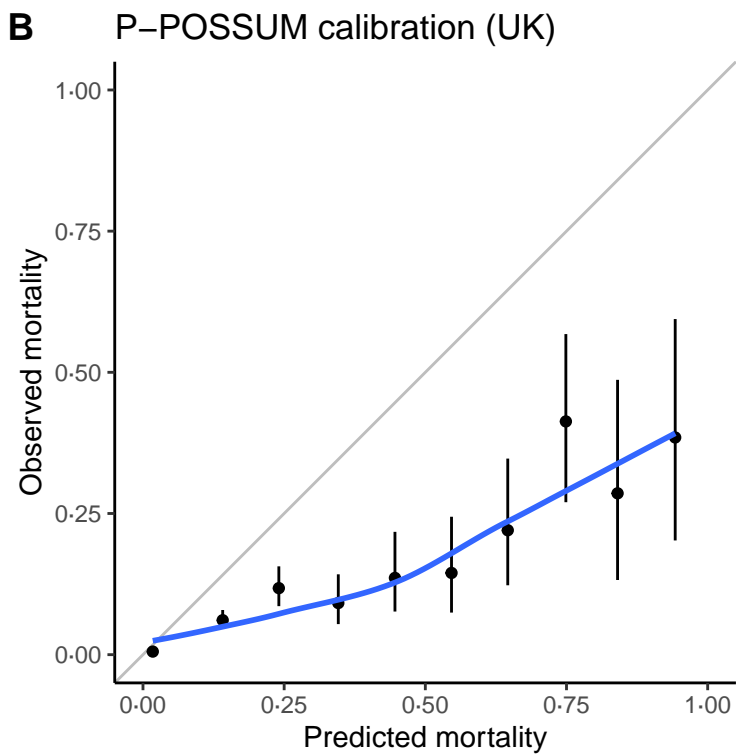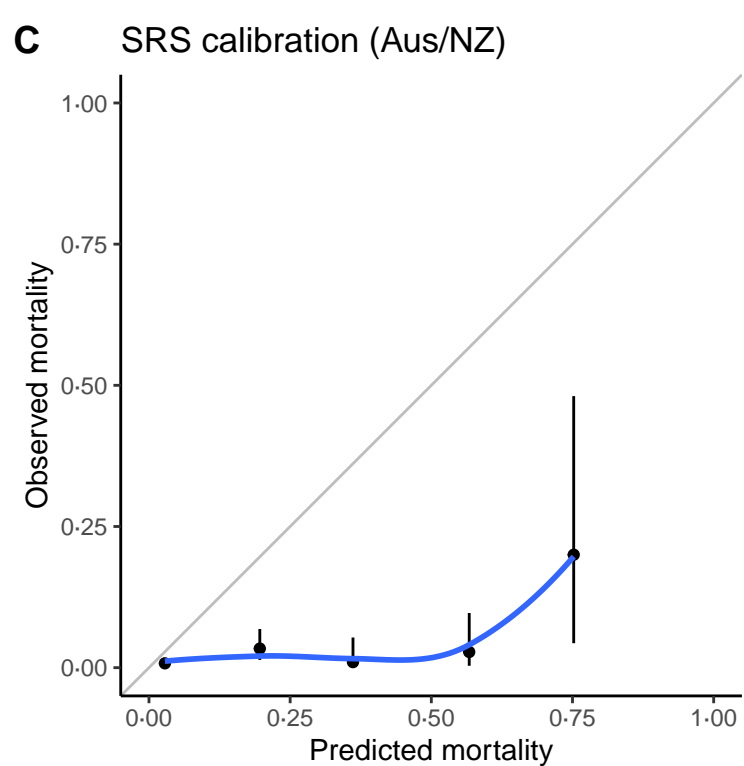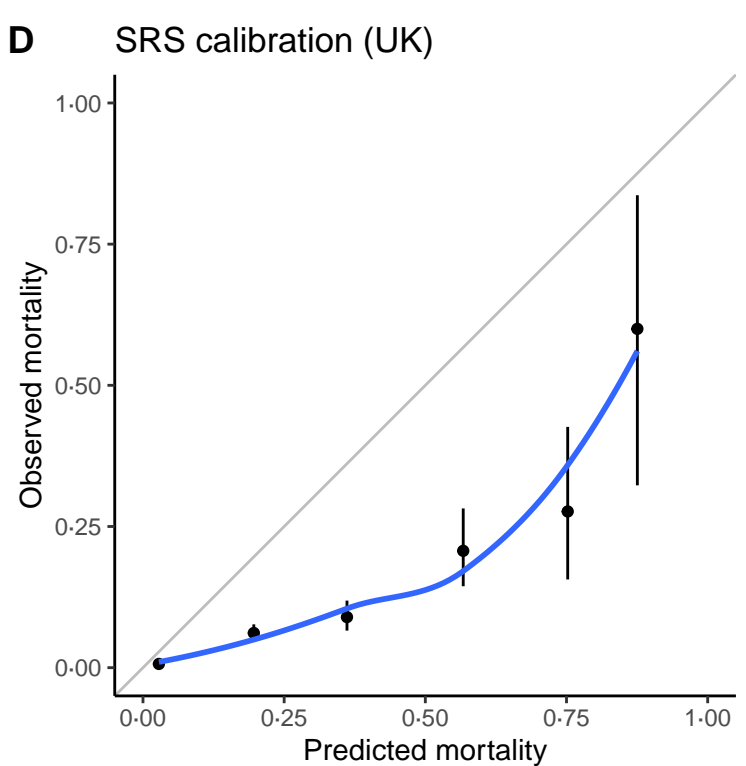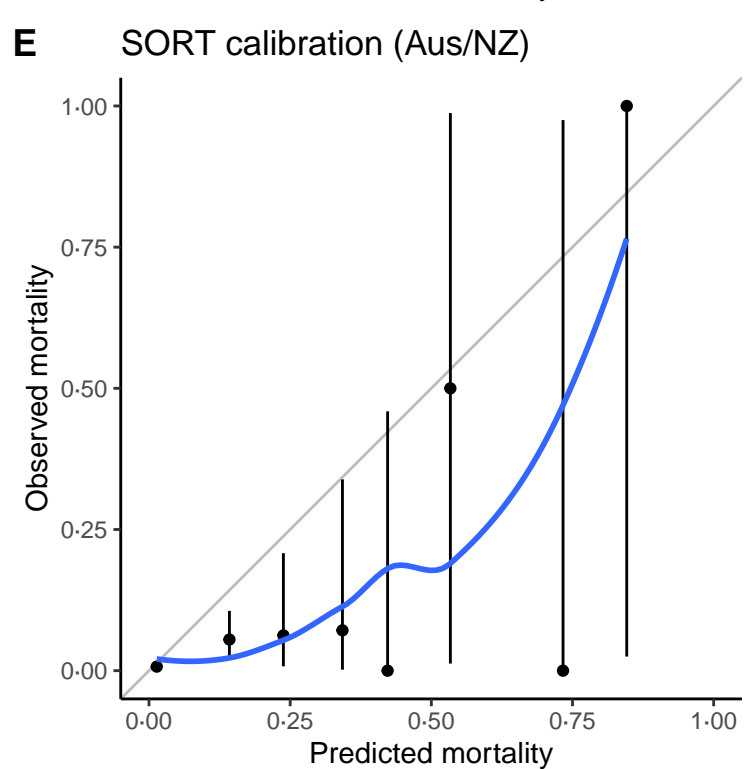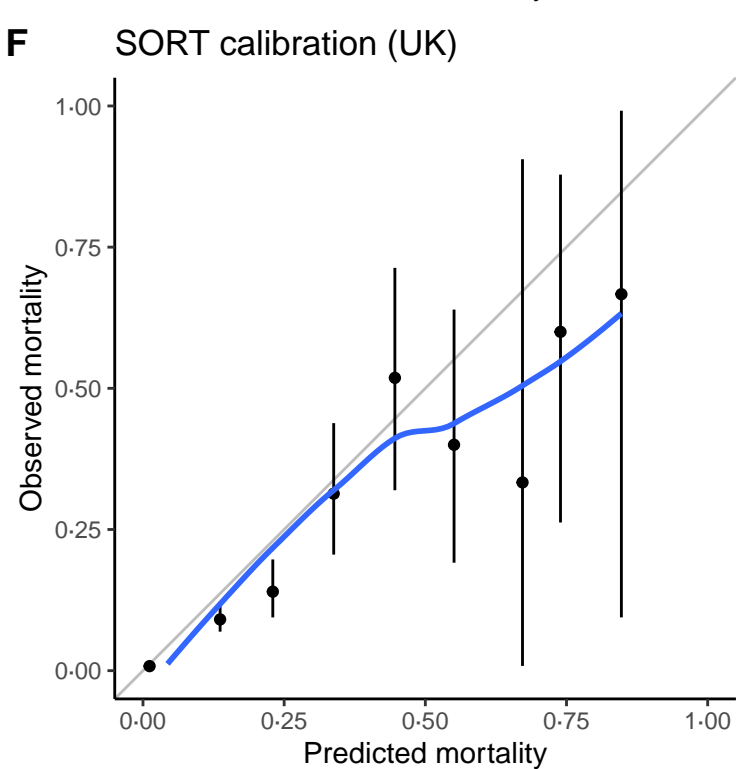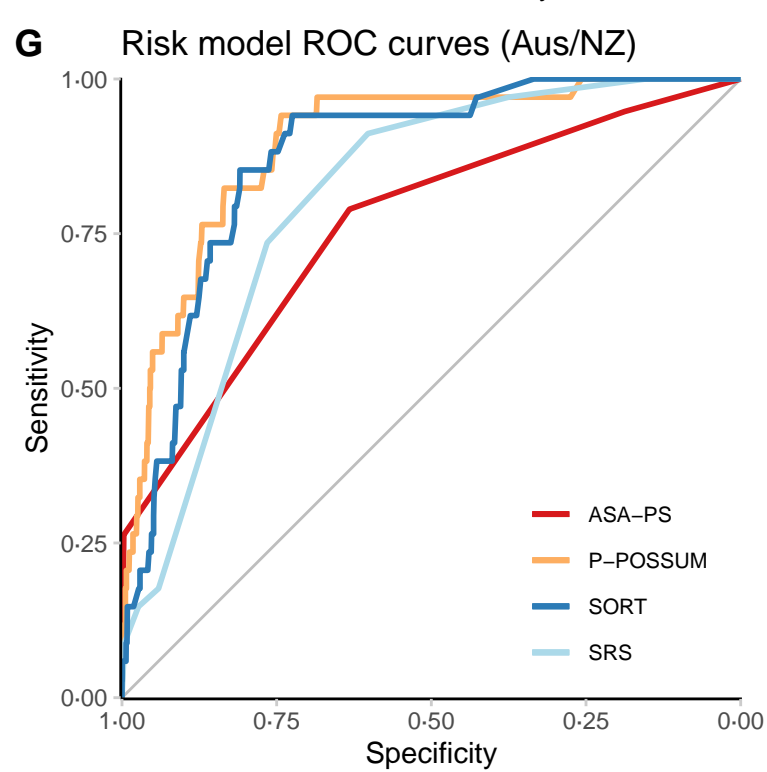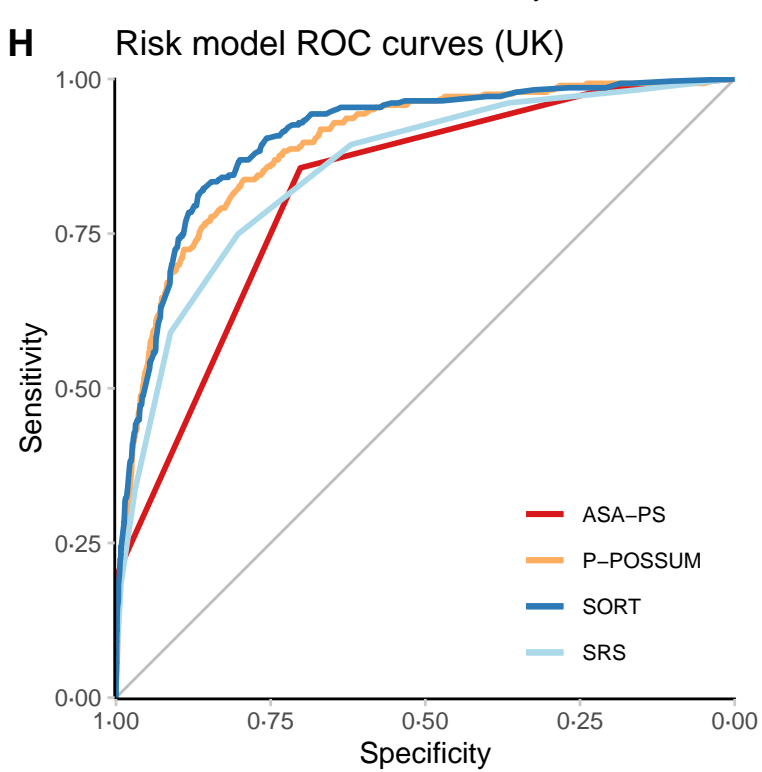

Supplement: S5 Fig — There was minimal difference between countries. ROC, Receiver Operating Characteristic. (PDF) [file pmed.1003253.s021.pdf]

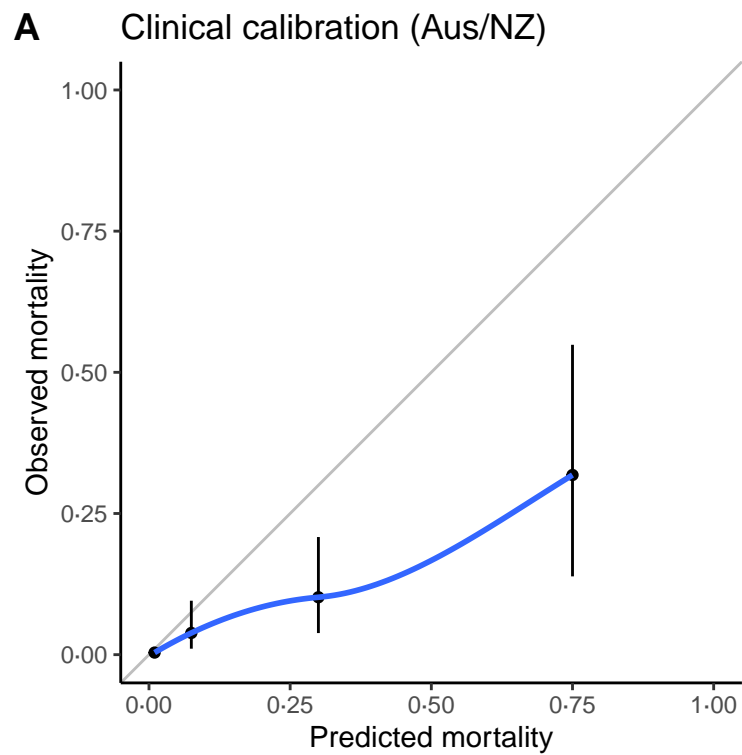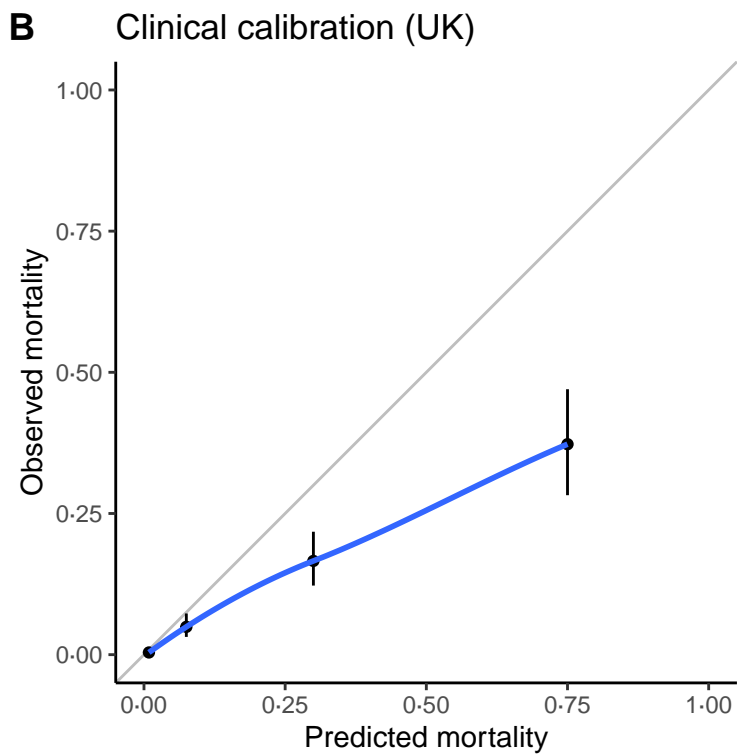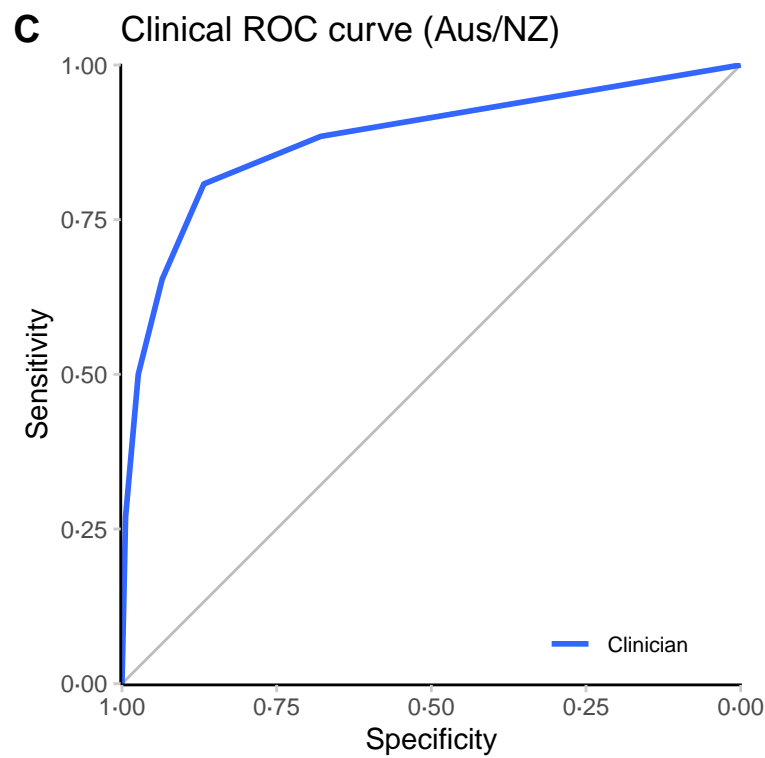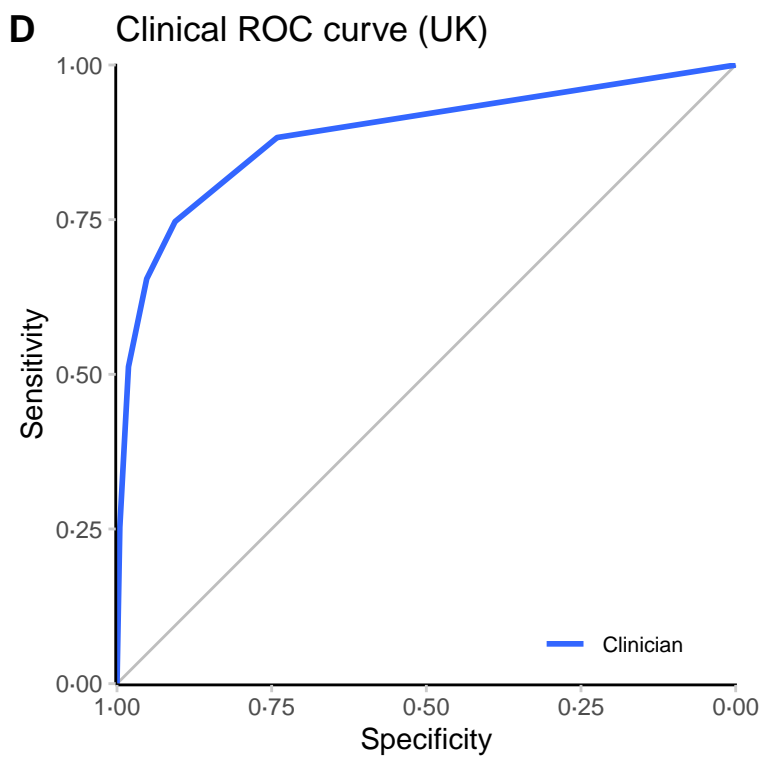

Supplement: S6 Fig — There was minimal difference between countries. ROC, Receiver Operating characteristic Curve. (PDF) [file pmed.1003253.s022.pdf]

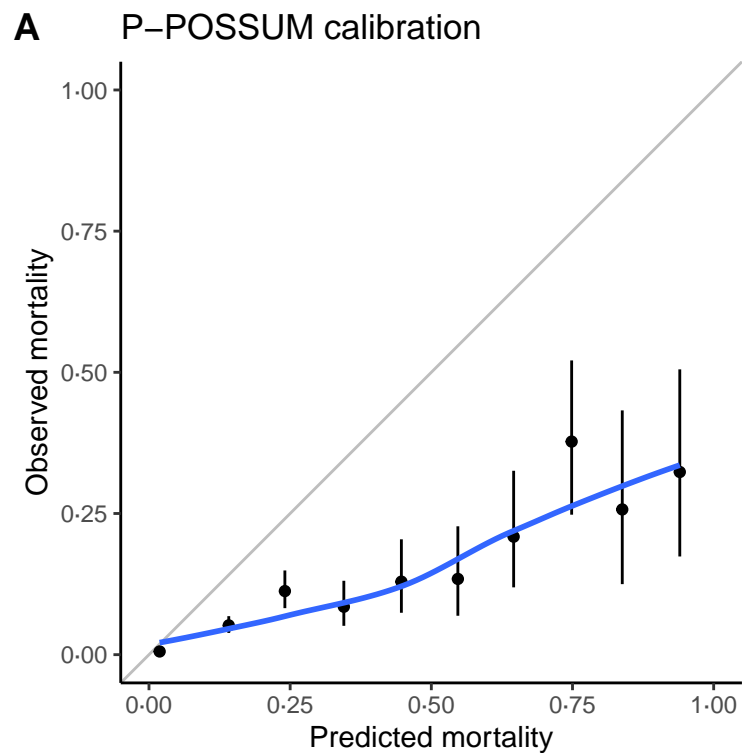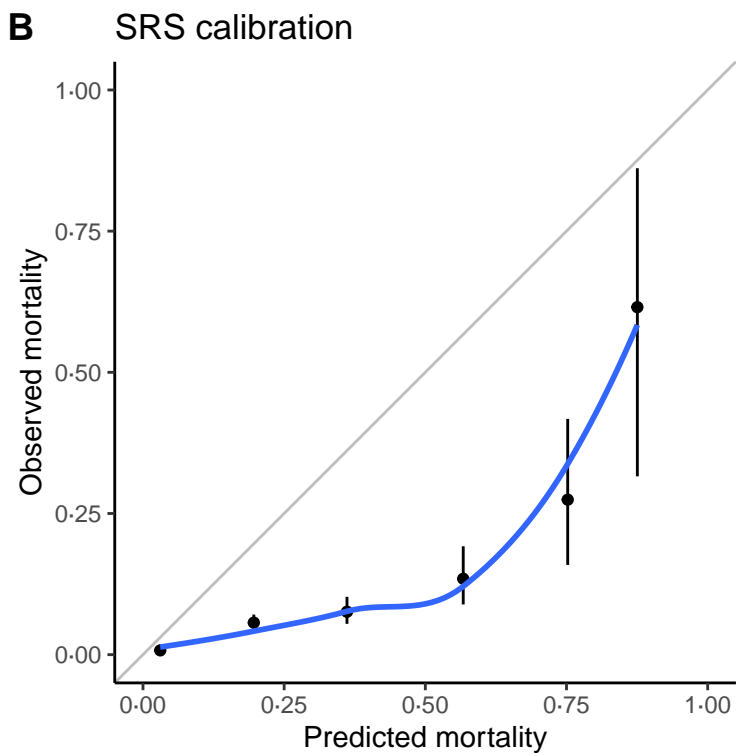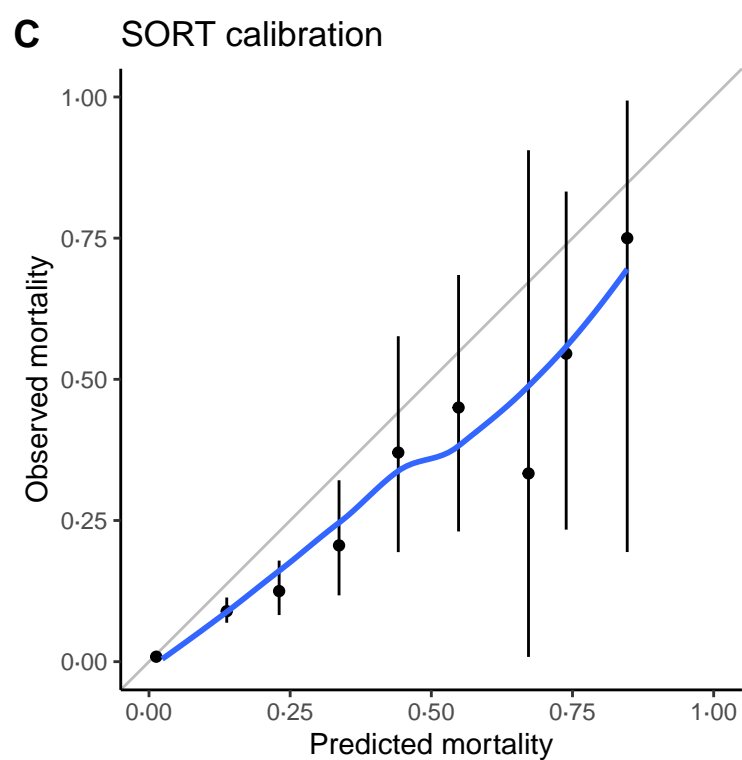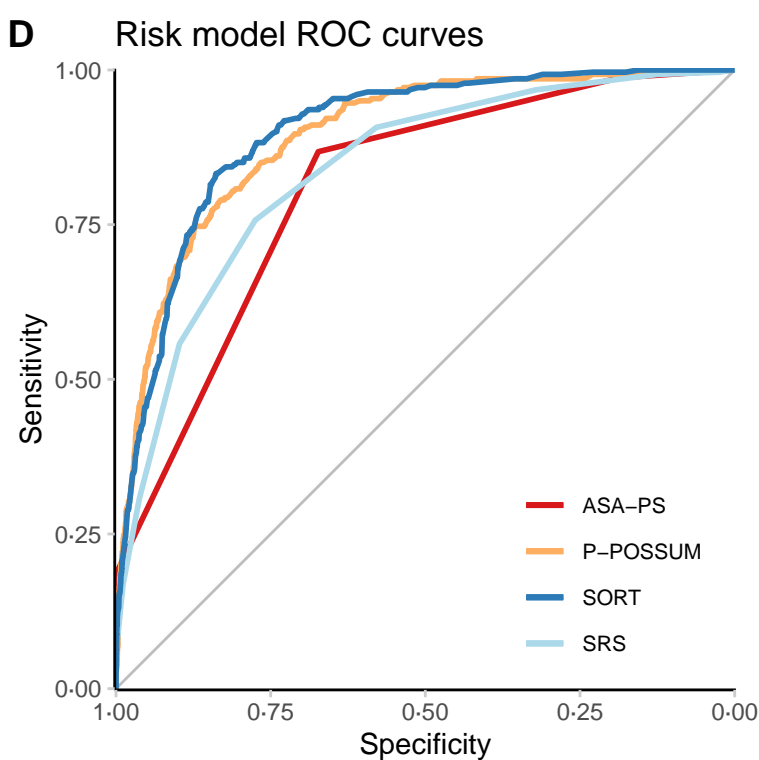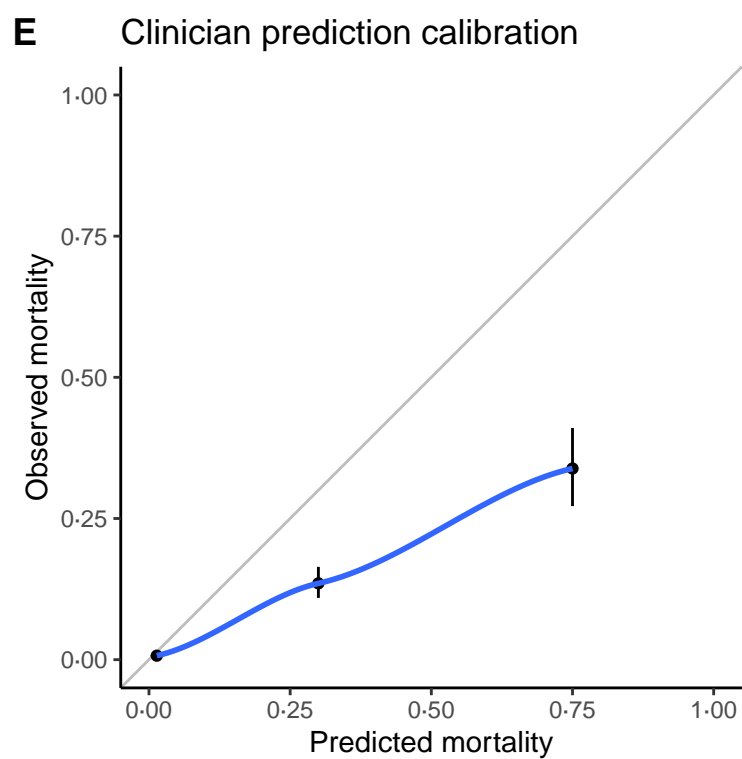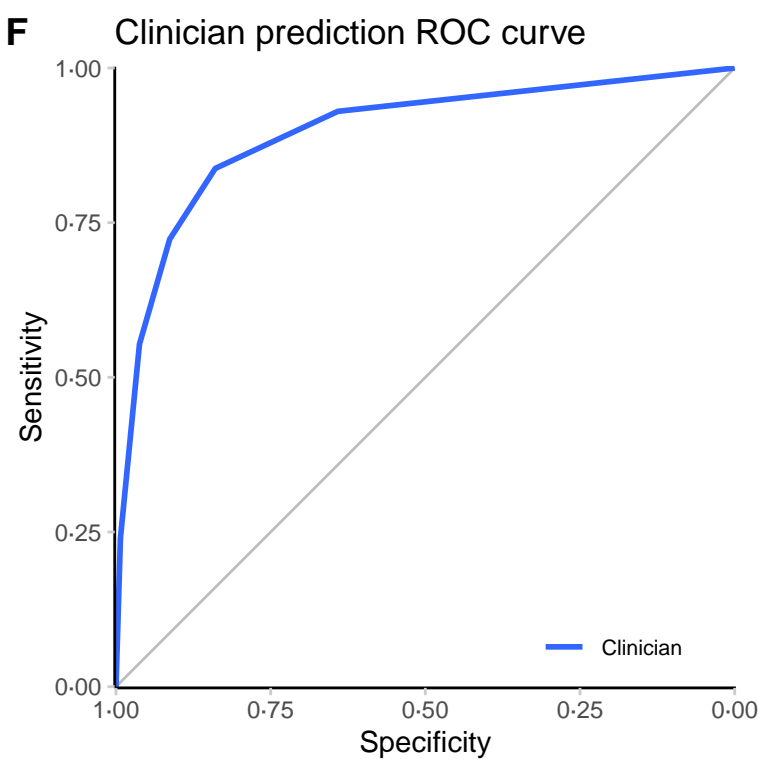

Supplement: S7 Fig — The AUROCs for P-POSSUM, SRS, SORT, and clinical assessments were 0.893, 0.838, 0.899, and 0.896 in this subgroup, respectively. AUROC, Area Under Receiver Operating Characteristic curve; P-POSSUM, Portsmouth-Physiology and Operative Severity Score for the enUmeration of Mortality; SORT, Surgical Outcome Risk Tool; SRS, Surgical Risk Scale. (PDF) [file pmed.1003253.s023.pdf]
