# Supplementary material for: Developing and validating subjective and objective risk-assessment measures for predicting mortality after major surgery: An international prospective cohort study
Source: PLoS Med. 2020 Oct 15;17(10):e1003253. doi: 10.1371/journal.pmed.1003253 (PMC7561094; doi:10.1371/journal.pmed.1003253)
Supplement: Attachment — Submitted filename: Reviewer responses SRM 01062020.docx [file pmed.1003253.s025.docx]

Dear Dr Gaynor-Brook

Many thanks for your correspondence and for giving us the opportunity to respond to the reviewers’ thorough evaluation of our research.

We are delighted to re-submit now, and are grateful for the extension which PLOS Med offered us in mitigation of our commitments to COVID19 related clinical work.

Our specific responses are **in bold** below and we hope that our manuscript now meets the standard required for publication in PLOS Med.

With best regards

Ramani Moonesinghe

on behalf of the authors.

-----------------------------------------------------------

Requests from the editors:

General comment: Please cite reference numbers in square brackets, leaving a space before the reference bracket, and removing spaces between reference numbers where more than one reference is cited e.g. '... postoperative complications [5,6].'

**Thank you – we have now done this.**

General comment: Please refer to a more specific part of your appendix to make clear to readers to what you are referring e.g. Figure S1, Table S1, etc.

**This has now been amended.**

General comment: Throughout the ms, please quote exact p values or "p<0.001", unless there is a specific statistical justification for quoting smaller exact numbers.

**We have increased the precision of p-values to 3 decimal places as requested.**

General comment: Please add line numbers to your revised manuscript.

**This has been done.**

Data Availability Statement: PLOS Medicine requires that the de-identified data underlying the specific results in a published article be made available, without restrictions on access, in a public repository or as Supporting Information at the time of article publication, provided it is legal and ethical to do so. Please provide the URL and any other details that will be needed to access data stored in the public repository.

**We have prepared a de-identified dataset for publication with the paper as supporting information.**

Title: Please revise your title according to PLOS Medicine's style, placing the study design in the subtitle (ie, after a colon). We suggest “Subjective and objective risk assessment for predicting mortality after major surgery: an international prospective cohort study” or similar

**Thank you - we agree the with the editors’ suggested title, and have amended it accordingly.**

Abstract Background: Please expand upon the context of why the study is important. The final sentence should clearly state the study question, and clarify the aims of the study e.g. to compare the accuracy of freely-available objective surgical risk tools with subjective clinical assessment in predicting 30-day mortality

**Thank you - we have clarified the importance of the risk prediction in guiding clinical decision making and resource allocations, and have reinforced the aims of the study in the background section of the abstract.**

Please combine the Methods and Findings components of your Abstract into one subsection with the heading Methods and Findings’

**This has been changed.**

Please include the study design, brief demographic details of the population studied (e.g. age, sex, types of surgery, etc) and further details of the study setting (e.g. what type of hospitals - secondary / tertiary care? Rural / urban? etc.), and main outcome measures.

In your abstract and elsewhere, please quote p values alongside 95% CI where available.

Please revise the sentence beginning ‘We included consecutive adults…’ to clarify. Please revise 'consecutive adults’ and ‘undergoing inpatient surgery for one week’

In the last sentence of the Abstract Methods and Findings section, please describe the main limitation(s) of the study's methodology.

**Thank you – we have made these changes**

Please begin your Abstract Conclusions with "In this study, we observed ..." or similar. Please address the study implications, emphasising what is new without overstating your conclusions.

Please tone down subjective language such as ‘highly accurate’

Please avoid vague statements such as " Clinicians can use this….", to instead highlight that ‘This may be of value in helping to stratify… ‘

**Thank you – we have made these amendments**

At this stage, we ask that you include a short, non-technical Author Summary of your research to make findings accessible to a wide audience that includes both scientists and non-scientists. The Author Summary should immediately follow the Abstract in your revised manuscript. This text is subject to editorial change and should use non-identical language distinct from the scientific abstract. Please see our author guidelines for more information: <https://journals.plos.org/plosmedicine/s/revising-your-manuscript#loc-author-summary> Please ensure to include a final bullet point under ‘What do these findings mean?’ to describe the main limitation(s) of the study.

**Thank you – we have done this.**

Introduction

Please explain the need for and potential importance of your study. Indicate whether your study is novel and how you determined that.

**Thank you we have done this**

Please define QI

**We have now defined this abbreviation.**

Methods

Please include the completed STROBE / TRIPOD checklists as Supporting Information and refer to the relevant supplementary files in your Methods section. When completing the checklists, please use section and paragraph numbers, rather than page numbers.

Please adapt "STROBE diagram" to "participant flowchart", or similar.

**We have amended this.**

Did your study have a prospective protocol or analysis plan? Please state this (either way) early in the Methods section. If a prospective analysis plan was used in designing the study, please include the relevant prospectively written document with your revised manuscript as a Supporting Information file to be published alongside your study, and cite it in the Methods section. If no such document exists, please make sure that the Methods section transparently describes when analyses were planned, when/why any data-driven changes to analyses took place, and what those changes were.

**This study was a planned analysis from the SNAP-2: EPICCS project, which is a wider body of research work and which had its protocol previously published. That protocol defined the aims, objectives and research questions of the project but did not include a detailed statistical analysis plan for the analyses in this manuscript. We have cited the published protocol paper in the Methods section.**

Please provide more detail on the hospitals included in the study e.g. secondary / tertiary care, how many from each country, etc.

**We have now provided more detail about the hospitals included in the study in the manuscript, under the Dataset subsection of the Methods.**

Please provide detail on the inclusion criteria, as this is notably missing from your supplementary material.

**We had included the inclusion criteria in the Supplementary Material, which unfortunately may not have been forwarded to the editor/reviewers. We have now included more details of our inclusion/exclusion criteria within the main manuscript under the Dataset subsection of the Methods.**

Please provide further detail on the institutional research and development department approvals for Northern Ireland, Australia and New Zealand as you have for the UK. This should include the names of the institutional review board(s) that provided ethical approval.

**Further details of the ethics and governance approvals received for the study in Australia and New Zealand have now been included under a new Ethical and Governance Approvals subsection of the Methods. As Northern Ireland is a constituent nation of the United Kingdom, ethical approval from the Health Research Authority applies in that country. We sought further local approvals for data acquisition without patient consent from individual Health and Social Care Trusts in Northern Ireland, which is now clarified in the text.**

Results

Please mention in the main text of your Results that 4,891 surgical episodes were excluded from analysis (as indicated in your STROBE diagram)

**This has been amended in the first paragraph of the Results.**

Please quote p values alongside 95% CI where available.

**Where there was a hypothesis test performed we have included p-values alongside 95% CIs. However, as some of the reported 95% CIs are measures of uncertainty around a particular estimate, e.g. the estimate of the area under the ROC curve, a p-value is not appropriate and therefore was not reported.**

Please refer to supplementary figures for the results presented for the sensitivity analyses.

**References to the supplementary figures and tables have now been included in the Results text.**

Please consider whether additional sensitivity analyses could be performed to at least partially address comments about inclusion of certain specialties that might bias the overall findings in a particular direction. Please ensure that it is made clear in your Methods that any such additional analyses are non-prespecified.

Table 1 - please revise ‘Xmajor’.

**“Xmajor” is a classification label name in the AXA-PPP severity system and stands for an ordinal level of surgical severity between Major and Complex. For this reason, we have left this label as it stands.**

Tables 3 & 4 - please define all abbreviations used in the table legend. When a p value is given, please specify the statistical test used to determine it in the table legend.

**This has been amended.**

Tables S3 & S4 - please define all abbreviations used in the table legend.

**This has been amended.**

Figure 2 - please define all abbreviations used in the figure legend Figure 4 - please provide a figure legend Supplementary Figures 3 and 4 - please provide a figure legend

**This has been amended.**

Discussion

Please present and organize the Discussion as follows: a short, clear summary of the article's findings; what the study adds to existing research and where and why the results may differ from previous research; strengths and limitations of the study; implications and next steps for research, clinical practice, and/or public policy; one-paragraph conclusion.

**Thank you – we have reviewed the discussion and made some additions, swapped around some text and believe that it now follows this structure.**

Please tone down subjective language such as ‘highly accurate’

**Thank you – we have done this.**

Please avoid vague statements such as " Clinicians can use this….", to instead highlight that ‘This may be of value in helping to stratify… ‘

**We argue that this is a matter of style and we feel that our language has been clear in the utility of our findings, e.g. in our final concluding statement, ‘Implementation of the SORT-clinical judgement model should lead to better clinical decision-making, and improved allocation of limited resources such as critical care beds, to patients who are most likely to benefit.’**

References

Please provide names of the first 6 co-authors for each paper referenced, before ‘et al’

Noting reference 11, please ensure that all references have full access details.

**Thank you – we have done this**

Supplementary Files

Please clearly label each supplementary file / table / figure with a distinct name and ensure that a reference is made to each component in the main text of the manuscript.

Please ensure that all components of your supplementary files e.g. inclusion criteria are included in your resubmitted manuscript.

**Thank you – we have done this**

**Comments from the reviewers:**

Reviewer #1: "Comparing subjective and objective risk assessment for predicting mortality after major surgery - an international prospective cohort study of 26,216 patients" evaluates three publicly-available objective risk tools against clinicians' subjective judgment on mortality prediction, and further integrates the best-performing objective risk tool with said subjective judgment to produce an even better-performing logistic regression model.

The prospective nature of the study, diverse demographics involved (over 26,000 patients from 274 hospitals in the United Kingdom, Australia and New Zealand) and open availability of the examined objective risk tooks (P-POSSUM, SRS and SORT) are particular strengths of the manuscript. Overall, the work seems to have the potential to broadly benefit surgical management practice. However, there are a number of points that might be expanded upon.

Firstly, on the comparison methods; the authors may wish to clarify their definition of objective vs. subjective for risk assessment procedures. In particular, the "objective" Surgical Risk Scale (SRS) appears to comprise a summation of ASA-PS scores together with the CEPOD and BUPA scores ("The surgical risk scale as an improved tool for risk-adjusted analysis in comparative surgical audit", Sutton et al., 2002), but the ASA-PS is itself considered "subjective" assessment (Page 8). Moreover, it is not clear whether CEPOD and BUPA are any less "subjective" than ASA-PS, from their descriptions.

**Thank you. We suggest that, as with many things in clinical practice, objectivity and subjectivity are on a continuum, and therefore a clear distinction can be difficult. CEPOD urgency is a defined scale of urgency and the BUPA procedure codes are explicitly defined surgical complexity categories used by private health insurers to allocate a cost to particular procedures, so we would suggest that these are objectively defined. We agree that ASA-PS is subjective to an extent, and although the ASA-PS grades are defined, their definitions allow some room for clinicians to deviate. Therefore to add clarity to our definitions, we have added a statement to the Methods section that reads, “In this study, we defined objective risk assessment as the use of a risk calculation model or equation or tool that supplies a prediction of risk on a probability scale.”**

Also, for P-POSSUM, it is noted that a number of biochemical/haematological parameters were missing in practice ("Missing data" section, Page 9), and in these cases, normal data was assumed/imputed. Moreover, there remained a small number of cases with further missing data beyond these bio/haemo parameters (as understood from "Following imputation, we performed a complete case analysis as we considered the proportion of cases with missing data in *the remaining variables* to be low [1.08%]"). This practice however seems problematic, in that close to half of the variables used for P-POSSUM appear to be biochemical/haematological (i.e. haemoglobin, WBC, urea, sodium, potassium). Simply assuming normal values for these variables would then appear to rob P-POSSUM of quite a bit of its utility, since these assumptions constitute unsubstantiated evidence towards better patient outcomes. The authors might clarify as to exactly how many cases were affected by missing data for P-POSSUM, and consider an analysis of only those cases where full data was available for P-POSSUM, for a fair comparison.

**As P-POSSUM applies scores to ranges of physiological/biochemical/haematological/other investigative values, e.g. a score of 1 for haemoglobin (Hb) in the range 130-160g/L, a score of 2 for a Hb in either ranges of 115-129 or 161-170mmHg, and so on. We did not impute values as normal when they were missing, but instead we imputed the score of 1, indicating the assumption that the patient’s physiological/bio/haem values were within the normal ranges. This distinction is important as we did not assume normal point estimates of the values, but instead assume that the patients’ values were in the normal range. This is how P-POSSUM is frequently used in practice when patients present for low-severity surgery. For example, increasingly patients would not have a chest radiograph (a component of P-POSSUM), or an electrocardiogram (also a component), if they gave no history of cardiorespiratory disease.**

**However, given the concern, we have added a 4^th^ sensitivity analysis to the study, performing the same analysis but only in those cases where full data was available for P-POSSUM, as the reviewer has suggested. This further sensitivity analysis did not find any differences in the performance of clinical assessment, P-POSSUM, SRS or SORT between the main study cohort and the subgroup with full P-POSSUM variables available.**

In general, the authors might consider summarizing the parameters & criteria used for the various objective methods as supplementary material.

Secondly, on the use of AUROC as a main quantitative assessment metric of the various methods - it is stated that the 30-day risk of death was predicted as one of six categorical responses, for all methods used (Page 7). Then, although the continuous net reclassification improvement statistic (NRI) is cited (Page 8), the authors might clarify in greater detail as to how this relates to the construction of ROC curves (i.e. Figure 2 & 3). For example, if the prediction is 0.5% and the patient indeed survives for 30 days, what sensitivity/specificity does this amount to as opposed to if the patient had not survived (we assume a binary outcome)? While readers might be familiar with the usual binary ROC formulation, the implementation of multiple categories might warrant more description.

**We apologise as we have not explained our analysis clearly enough. The 6 categorical responses were not the prediction obtained for all the methods used, instead they were only the prediction from subjective clinical assessment. The formulation of the ROC curves in all the circumstances in our study still refer to the binary outcome (dead/alive at 30 days). The objective risk assessments give a continuous probability prediction of a binary outcome which maps onto the ROC curve. NRI and ROC curves are not interrelated, they are separate and different methods of assessing performance. We have provided an explanation of ROC curves for when the predictions are ordinal categorical, which is how the AUROCs are calculated for ASA-PS and for clinician assessments, to increase the clarity of this.**

Further on the categorial prediction, the calibration graphs in Figures 2 and 3 appear to suggest that the various methods output different numbers of point estimates. For example, P-POSSUM has 10, SRS has 6, SORT has 9 and clinicians have 4 (as opposed to the six categories implied in the Dataset section). While it seems that a greater number of point estimates improves the level of detail of the corresponding ROC curve, the effect seems exaggerated for SORT and SRS; in particular, the ASA-PS ROC curve in Figure 2 seems to be a piecewise construction from about 3 datapoints, while the SRS curve likewise seems to be piecewise constructed from about 6 datapoints. However, the SORT and SRS curves have much finer detail (i.e. have an independent value for each 0.01 change in specificity or less), despite only having slightly more point estimates than SRS. The authors might wish to explain this discrepancy.

**Thank you – please see the above response. The predictions are not categorical for all models; the clinicians’ assessments are the only categorical predictions (which in themselves fall in the probability range of 0 to 1). For P-POSSUM, SRS and SORT, predictions are continuous variables: the probabilities of a binary outcome. The reason why the calibration graphs for clinicians has 4 points, is that in turning a categorical prediction into a point estimate, the lower categories of <1%, 1-2.5%, and 2.6-5% are so closely situated on the 0 to 100% scale in the graph that the points effectively overlap, and because these are the categories where most of the predictions are distributed, the confidence intervals are also extremely tight that they are covered by the dot on the plot.**

Thirdly, while there is detailed analysis of the aggregate statistics for the various methods (including on a high-risk patient subset), an additional inter-model analysis at finer granularity would seem to be appropriate. In particular, do the various objective and subjective models tend to agree/disagree on specific patients, and in cases where they strongly disagree (i.e. one method predicts a very low mortality risk, while another method predicts a significant risk, for the same patient), what are the factors that might have led to this disagreement? Such an analysis would help to determine whether the choice of particular models (e.g. the combined logistic regression model vs. SORT alone) is strictly beneficial for all patients (strictly dominating method), or if it may shift the risks of inaccurate prediction from one group of patients onto another group.

**We would like to seek clarification on the reviewer’s request. Does the reviewer want us to select individual patient cases/scenarios and see how these patients would score on the different scoring systems? Our concern with this approach is that it could show selection bias as we would be handpicking patients from the 26k patient pool. We have a concern that this is not a means of validating risk prediction models and departs from the guidance laid out by TRIPOD [Moons KGM et al. Transparent Reporting of a multivariable prediction model for Individual Prognosis Or Diagnosis (TRIPOD): Explanation and Elaboration. Annals of Internal Medicine. 2015 Jan 6;162(1):W1.]. The reviewer’s suggestion may be more suited to case reports of particularly unusual clinical scenarios where risk-prediction models may fail as certain variables might not be used in model construction (“Black Swan” events, which models are unable to predict). For these reasons, unless we have misunderstood the request, we would prefer not to add this analysis.**

A confusion matrix of the six prediction categories vs. binary outcomes at a reasonable ROC operating point for each method would also be illuminating.

**Thank you. We have a concern because the our interpretation of the risk prediction literature is that we should not be picking a single cut-off ROC value based on ROC curve analysis [Moons KGM et al. Transparent Reporting of a multivariable prediction model for Individual Prognosis Or Diagnosis (TRIPOD): Explanation and Elaboration. Annals of Internal Medicine. 2015 Jan 6;162(1):W1.]. However, we have produced a confusion matrix at the 5% mortality threshold which is recommended in two sets of UK guidance as being the minimum safety point for referring patients for postoperative critical care. This is a risk threshold advised by expert panel consensus based on both resource availability and perceived benefit to the patient for critical care admission post-surgery. This confusion matrix has been added to the Supplementary Material as a table.**

Fourthly, the authors may wish to comment further on the practical implications of accurate mortality prediction (it is noted in the Discussion section that accurate prediction may result in fewer inappropriate admissions to critical case, though there remains a lack of "real-world" clinical uptake); will such predictions be used by clinicians/patients in deciding whether to commence surgery? Indeed, given that mortality estimates were made by the perioperative team beforehand for participating patients, did these estimates have any impact on the treatment offered (i.e. the abovementioned reduction in inappropriate admissions)?

**Thank you – we have added a sentence in the discussion about this.**

Finally, while perhaps somewhat out of the scope of this manuscript, the authors might consider exploring data-centric machine learning methods in the future (i.e. train models directly from the available patient demographic features)

**We briefly touch on the idea of incorporating machine learning methods in the Discussion, “Modern approaches to risk assessment using machine learning provide promise for automation of risk prediction and incorporating data and calculations which clinicians may subconsciously consider when making subjective decisions – however, even these methods do not substantially outperform our simpler approach, and are currently limited by recruitment biases and lack of availability.”**

Reviewer #2: In this international prospective cohort study, the authors adress the very important issue of preopeative risk assessment for mortality.

The article is clear an well written, but there is an important methodological question that have to be considered.

1) It is certainly a very good point to test the subjective risk assesment. However a main limitation is that the physicians could not have used objective assessments scores to help them answering the question proposed. These cases should have been excluded for all analysis initially (flowchart 1). Additionally, patients for whom ASA score was used were kept in the other analysis and classified as belonging to the `subjective analysis `group. Altough ASA score is the oldest and has a lot of subjectivity in itself, it could not be considered that no score was used. Also, the numbers in Flowchart 1 and Table 2 are a bit confusing (23540 patients with clinical judgment only and 9928 with ASA score in Table 2 and in flowchart 21325 patients inluded). The main analysis should have been done only with patients classified by the clinical judgment according to the answer of the question proposed and not using any additional tool, including ASA.

**As in our response to Reviewer 1, we agree that what is considered subjective and objective risk assessment is somewhat subjective in itself and there is no consensus. We have taken the decision to define an objective score as one that produces a probability prediction and have clarified this in the Methods. We had originally intended to restrict our analyses in the second and third parts of the paper to clinicians who have not used ASA. We spent a long time among the authorship group discussing the “subjectivity” of ASA. We concluded that although ASA has definitions associated with it, there is evidence of substantial inter-rater variability in ASA grading between clinicians [Tollinche LE, Yang G, Tan KS, Borchardt R. Interrater variability in ASA physical status assignment: an analysis in the pediatric cancer setting. J Anesth. 2018;32(2):211‐218. doi:10.1007/s00540-018-2463-2]. For that reason, we elected to keep it within the subjective risk assessment group. We hope that this response satisfies the reviewer’s concerns.**

**We apologise that the numbers are confusing; this is because clinicians were able to select multiple choices when considering which tools they used to reach their decision about clinical risk assessment. So, for example, in 23540 patients, the clinicians ticked either clinical judgment only, or clinical judgment and any of the others; 9928 are those who ticked ASA-PS, regardless of whether they ticked clinical judgment or any of the other methods; 21325 are those who ticked clinical judgment AND/OR ASA-PS, minus those with missing values. The external validation as performed using 26216 patients. For different parts of the analysis, we used different subsets of the data. We have tried to make this clearer in the text – the first paragraph of the results.**

2) It is stated in methods that data imputing was done to the laboratory values that were missing to the POSSUM score. Please provide how many imputation was performed. Additionally, assuming that the values are normal in patients undergoing elective surgery without a blood withdraw available, that may not be accurate for emergency patients, for whom there was no time to perform a blood test.

**Thank you. Please see our reply to Reviewer 1 for the same issue. We imputed normal ranges which carry a physiology score for P-POSSUM, and we did not impute single values. To examine whether our findings are robust to only including cases with full P-POSSUM variables, we performed an additional sensitivity analysis as suggested by Reviewer 1, which also addresses Reviewer 2’s comment.**

3) Although there is no consensus of what is the best risk stratification tool, in the discussion the authors mentioned that the limitation of the NSQIP risk calculator was never validated outside the US. This would have been a good opportunity to do so because, although it involves more variables, it is online available for free and could be easily implemented in an electronic chart, for example.

**Thank you. We agree that this is a missed opportunity although as NSQIP do not publish**

**their coefficients, the only way to validate their calculator in our cohort would have been to manually enter every patient into their public-facing website, which we did not have the resource to do.**

Reviewer #3: Dear authors

Thank you to provide me the opportunity to read your manuscript, it reports a comparison of objective and subjective predictive tools to predict postoperative mortality after major surgery.

While the objectives of the study are interesting and would have some potentially important clinical applications, several major limitations limit the interpretation of the observed results.

**We thank the reviewer for their time.**

My key suggestions to improve the impact of this study are:

To select preoperative (not P-POSSOM) scores that do not already include a subjective component (neither SORT nor SRS).

**Unfortunately, we are limited by the data we collected and therefore unable to evaluate any other models or methods.**

To not attend to create a universal predictive tool working for any type of surgeries.

**Thank you. Respectfully, the aim of our study was to do exactly this. Clinicians are faced with decisions about how to allocate resources (particularly critical care beds) on a daily basis which require comparisons to be made between patients having different types of surgery. One of our key objectives was to develop methods which would assist clinicians in such decision-making.**

To better describe the subjective score introduced: inter-rater variability, distributions and eventually to reduce the number of strata to limit variability.

**Thank you. Unfortunately, we are not able to do this. We believe that the reviewers’ question is interesting but it differs from our main research question which was to compare accuracy of the clinical team’s subjective assessment with widely available objective tools.**

To emphasize the results provided by the decision curves rather than focussing on AUC ROC in models with poor calibration.

**Thank you. It is reassuring to us that the reviewer is satisfied we used decision curves. However, while decision curves provide useful information for decision-making, TRIPOD still regards discrimination and calibration as the mainstays of assessing clinical prediction tool performance. We have therefore reported all three in our paper.**

I summarized the most important methodological concerns below

**Thank you. Many of this reviewer’s concerns relate to the inclusion of obstetric patients in this cohort; therefore, we have removed this group of participants. This has not yielded any major differences in our findings.**

1 - Population: The study population includes a non-representative mix of cases including some surgical specialties that are associated with high postoperative mortality and in which general predictive tools don't work well (i.e cardiac surgery - 3.9% of the cohort, 5.7% of the observed deaths).

**Thank you. We would like to offer a gentle rebuttal to this assessment. We recruited consecutive patients for a week without consent thereby limiting the bias in our sample substantially, compared with similar studies. Our aim was to capture the whole spectrum of risks across a heterogenous cohort of patients.**

**We would like to politely refute the idea that cardiac surgical patients are particularly high-risk in comparison to the rest of the population under study. In our cohort, although the cardiothoracic patients represented 3.9% of the cohort and 5.7% of the observed deaths, they had an equivalent 30-day mortality risk to GI surgery patents (Cardiothoracic 19 deaths out of 1041 cases = 1.83% mortality; GI surgery 88 deaths out of 4494 cases = 1.96%).**

**We have undertaken new sensitivity analyses in specific surgical sub-groups in order to reassure readers and reviewers that the SORT-clinical judgement model has validity across different specialties. (supplementary material sensitivity analysis 6)**

The objective of the study is to evaluate the predictive performances of scores already described elsewhere. Therefore, we are not seeing representative samples and we are simply evaluating the performances of some predictive models. Unfortunately, the combination of the clinical prediction and the other scores is dependent of the study population case-mix, because a new model has been fit. Whether this combined model is working on other populations with a different case-mix is unknown and must be discussed properly as an important limitation.

**Thank you. As with all internal validation studies, this is a limitation and we have discussed this point.**

2 - Population (2): The inclusion of obstetric is a usual mistake we can find in similar studies. It increased the number of patients in the cohort, but it is not associated with any deaths. Therefore, it dropped the average apparent mortality (no deaths after obstetrics in the study we are discussing today) and make the methodological tools used to compare the predictive models not accurate and potentially biased. Further, with no deaths and a clearly identified subgroup (which is included in the calculation of the scores), the inclusion of OB patients artificially increases discrimination performances but is not clinically relevant.

**We have considered the possibility that we are studying a cohort with overall low risk, and have therefore performed a sensitivity analysis with a higher-risk subgroup of around 13000 patients, among which included patients with any of the following: : intermediate-to-high risk surgery (intra-peritoneal, intra-thoracic, or major vascular surgery); history of coronary artery disease; history of heart failure; history of previous cerebrovascular disease; history of diabetes mellitus require drug therapy; preoperative renal insufficiency; history of peripheral arterial disease; history of hypertension; age ≥70 years.**

**We reiterate that we have now dropped the obstetric cohort.**

Which clinician(s) would use the same score to predict accurately the outcome of OB and cardiac patients?

**Thank you. Please see our responses above about the importance of having a single system to evaluate a surgical population in everyday clinical practice. Further, we would argue that clinicians use their subjective assessment regardless of the specialty of the patient they are treating in a similar way, and furthermore that the type of surgery forms part of the decision making which clinicians undertake subjectively. We concede that none of the obstetric patients suffered a 30-day mortality outcome in the study and therefore we should not be assessing a mortality prediction tool with this population included. We have therefore rerun our analysis with the obstetric cases excluded, and added an additional sensitivity analysis with the obstetric cases included. This has not yielded any major differences in our findings. We have also undertaken further sub-group analyses (supplementary material, sensitivity analysis 6) which confirms the good discrimination and calibration of the SORT-clinical judgement model in individual surgical sub-specialties. Only in vascular surgery (the smallest surgical cohort) did the calibration fail the Hosmer-Lemeshow assessment.**

3 - Major methodological mistake:

We are dealing with low-frequency primary outcome (i.e. 30d mortality = 1.2% - 317 deaths in 26,616 patients).

P-POSSUM, SRS and SORT showed terrible calibrations in figure 2, where we are observing dramatic overestimations of the risk of death (The calibration curves are way down in the lower right part of the calibration plots). This common methodological concern is poorly detected with ROC curves. Combining infrequent outcomes and poorly calibrated predictive models constantly produce very high AUC while models have no clinical values. That is what we observed here - and in many other studies dealing with similar population characteristics. including OB patients amplified the phenomenon.

**Perioperative mortality is low in general in high-income countries, but we continue to need to identify the small cohort of patients at higher risk within the larger low-risk population. Our data match those of large administrative datasets in terms of population characteristics and mortality outcomes (e.g. Abbott, Fowler, Dobbs et al: Frequency of surgical treatment and related hospital procedures in the UK: a national ecological study using hospital episode statistics; BJA 2017).**

**We acknowledge that calibration with P-POSSUM, SRS and SORT was poor and indeed to evaluate this was a key objective of our study so we have reported this clearly. These tools are generally used in clinical practice and this study highlights some of their flaws. We also supplemented our external validation of objective risk tools with looking at how well clinicians themselves are calibrated, also showing that clinicians in general have a poorly calibrated predictive ability. We contend that we did not just rely on calibration and discrimination in our assessments (these are the mainstays of validation according to TRIPOD), but have also included continuous Net Reclassification Improvement and Decision Curve Analysis in our statistical analysis. Therefore, we have tried to examine each objective tool, and subjective clinician judgement in as rigorous a manner as possible.**

4 - Major methodological mistake (2):

SORT : Truly preoperative, include ASA (subjective and wide inter-rater variability), fine description of the surgical procedures

P-POSSUM: More objective risk score with less inter-rater variability, but includes intraoperative variable not available SRS preoperatively

SRS: Truly preoperative, include ASA (subjective and wide inter-rater variability) and a subjective stratification associated with the planned surgery.

The new Subjective assessment introduced in this study: Preoperative, very subjective, 6 categories, somewhat like ASA. NO quantification of the inter-rater variability which is suspected to be very high in the not extreme categories.

**We agree that inter-rater variability exists with subjective assessment. However, this rating was a consensus of the clinicians in the operating room at the start of surgery, and therefore subject to less individual bias.**

2 of the 3 scores evaluated already include a clinical subjective evaluation of the preoperative patients' characteristics (i.e. ASA with all the limitations we know)

1 score includes intraoperative characteristics which make the comparison impossible.

**The way P-POSSUM is used preoperatively is by clinicians inputting the intraoperative variables they expected to find (preoperative P-POSSUM) and then revising it after the operation with the new intra-operative information (postoperative revised P-POSSUM). This approach is widespread in the UK and was for a long time a standard practice as part of the National Emergency Laparotomy Audit (NELA,** <https://www.nela.org.uk/>**), we thus felt it was justified to assess P-POSSUM in this way against the other risk tools.**

**Furthermore, as we included P-POSSUM values with intraoperative findings in our assessment and still found that it underperformed, we believe it was fair to conclude in our study that P-POSSUM should probably be superseded by other newer tools.**

The authors finally combined SORT and the clinical prediction they introduced. They demonstrated in the study population (after model fitting) that the predictive performance are somewhat better, and that the calibration is way better (they fit a new model...) SORT already includes ASA, how does ASA interacted with the clinical prediction which look very similar.

While it is recognized that the inter-rater variability of ASA is huge, what do we know about the inter-rater variability of the clinical prediction described here?

**We unfortunately did not quantify the inter-rater variability of subjective clinician assessment in our study, but as aforementioned, the way the question was asked at the start of surgery was “What is the estimate of the perioperative team of the risk of death within 30 days?” which seeks a consensus of the opinions in the operating room about the patient’s risk. We assert that this reduces the chances that there was wide inter-rater variability.**

5 – The use of decision curves is a very strong methodological part of this study, Unfortunately, their description and interpretation are somewhat neglected compared to useless ROC comparison. The reviewer strongly recommend to expand the description , interpretation and discussion of the decision curves which actuaaly provide the information that clinicians are seeking.

**Decision curves were incorporated as part of the overall comparison of the different models and is not the mainstay of validation according to TRIPOD. We therefore did not focus on this in our interpretation. However, given the Reviewer’s comments, we have added to the DCA interpretation in the text.**

Some specific comments:

Page 18 line 1:

"The SORT was the best-calibrated of the pre-existing models, however all over-predicted risk (Figure 2A-2C; Hosmer-Lemeshow p-values all "

HL test is not an appropriate approach to evaluate calibration in large cohorts where the frequency of the outcomes is low (See Tripod statements). Further a HL stat probability lower than 0.10 suggests mis-calibration. In the figure 2, HL probabilities are all lower than 0.0001. This somewhat confirms the visual analysis of the curves suggesting that the calibration of these scores is terrible (results already widely describe elsewhere)>

**We agree and included HL statistics here only for completeness. We agree that we are already able to tell that the calibration of these scores is poor, particularly for P-POSSUM and SRS.**

Page 18 line 6:

"All models exhibited good-to-excellent discrimination (Figure 2D; AUROC SORT=0.91 (95% confidence interval (CI): 0.90-0.93); P-POSSUM=0.90, (95% CI: 0.88-0.92); SRS=0.85 (95% CI: 0.83-0.88)."

High observed discriminations are the consequence of:

1/ poor calibration of the models and

2/primary outcome #1.2%.

**This comment has been addressed above. We have also addressed this in our discussion where we write, “We recognise some limitations to our study. First, models predicting rare events may appear optimistically accurate, as a model that identifies every patient as being low risk of mortality, in a group where the probability of death approaches 0%, would almost always appear to be correct.” Unfortunately this is an inherent to risk prediction research in the field of perioperative medicine where historical advances to surgery and anaesthesia has resulted in surgery being very safe in general.**

More importantly, no discrimination should be interpreted with such a poor calibration.

**We agree, and emphasise that we did not interpret any of the risk tools using discrimination in isolation. For the reasons we have described above, we present a complete assessment of performance using multiple measures, including calibration, NRI, DCA, in addition to discrimination. Furthermore, we provided several sensitivity analyses to demonstrate that our findings are consistent across multiple population groups.**

Figure 5:

This figure looks great, but some confidence interval would probably show that there are some major overlaps between the clinical prediction strata

**We agree there will be overlap in some of the confidence intervals for the ROC curves. By overlaying the curves and showing the 95% confidence intervals in the AUROCs in a table we have enabled the reader to make an assessment of the performances in conjunction with all the presented information.**

Thank you again for your work and I hope my comments would be useful in your work.

There is a true need for preoperative stratification/predictive tools and this work has the potential to fill a part of this need

**Thank you for this rigorous review of our work.**

Yannick Le Manach MD PhD

Reviewer #4: The manuscript entitled „Comparing subjective and objective risk assessment for predicting mortality after major surgery - an international prospective cohort study" was reviewed. In this study, the authors aimed to compare subjective and objective risk assessment tools, which are utilized in predicting the probability of the postoperative mortality. The study was a well-designed, prospective, multicentric, observational study, with great effort to reduce the bias as much as possible, in terms of recruitment phase and data analysis. However, I there are some concerns regarding the manuscript, which should be addressed.

Comments:

1. In a recent study, Chan et al. calculated the derivation and validation cohorts for mortality using the Combined Assessment of Risk Encountered in Surgery (CARES) surgical risk calculator, which is also comparable to the present study outcomes. The authors should also discuss the findings of this study.

**Thank you for highlighting this study – we have included it in our discussion.**

2. How did the authors define the needed cut-off values for classification of preoperative estimation of mortality risk?

**These thresholds were decided by expert consensus within the study steering group (comprising expert representatives from the Royal College of Anaesthetists, Faculty of Intensive Care Medicine, Intensive Care Society UK, Royal College of Surgeons (England), British Association of Critical Care Nurses, a patient lay representative, and the study authors. We have added clarification within the Methods text.**

3. I would suggest the authors to perform a subgroup and validation analyses by classifying the patients based on the type of surgery.

**Thank you – we have done the suggested sub-group analyses by surgical specialty (supplementary material, sensitivity analysis 6) which shows that even in these sub-groups, the SORT-clinical judgement model retains excellent performance (discrimination in all specialties, calibration in all but vascular surgery).**

4. How would it be possible to combine the two objective and subjective assessments in clinical practice. The combination of the tools has improved the outcomes for sure, but authors did not suggest any combined approach to establish an estimation using both risk assessments simultaneously.

**Thank you. We have made it clearer that our intention is to publish a calculator which enables clinicians to enter their assessment of risk and for this to be combined with the SORT, using the model we have developed, to produce a mortality risk estimate.**
